# Spatiotemporal Mapping of Efficient Chiral Induction by Helicene‐Type Additives in Copolymer Thin Films

**DOI:** 10.1002/anie.202203075

**Published:** 2022-06-23

**Authors:** Marius Morgenroth, Mirko Scholz, Laure Guy, Kawon Oum, Thomas Lenzer

**Affiliations:** ^1^ University of Siegen Faculty IV: School of Science and Technology Department Chemistry and Biology Physical Chemistry 2 Adolf-Reichwein-Str. 2 57076 Siegen Germany; ^2^ Univ. Lyon ENS de Lyon CNRS UMR 5182 Université Claude Bernard Lyon 1, Laboratoire de Chimie 69342 Lyon France

**Keywords:** Circular Dichroism, Circularly Polarized Luminescence, Supramolecular Chirality, Thin Films, Ultrafast Spectroscopy

## Abstract

We observed efficient induction of chirality in polyfluorene copolymer thin films by mixing with helicene‐type chiral additives based on the dibenzo[*c,h*]acridine motif. Images obtained from circular dichroism (CD) and circularly polarized luminescence (CPL) microscopy provide information about the chiral arrangements in the thin films with diffraction‐limited resolution. The CD signal shows a characteristic dependence on the film thickness, which supports a supramolecular origin of the strong chiral response of the copolymer. In particular, we demonstrate the discrimination between films of opposite chirality based on their ultrafast transient chiral response through the use of femtosecond broadband CD spectroscopy and a newly developed setup for transient CPL spectroscopy with 28 ps time resolution. A systematic variation of the enantiomeric excess of the chiral additive shows that the “Sergeants and Soldiers” concept and “Majority Rules” are not obeyed.

## Graphical abstract

A special class of helicene‐type additives, which are straightforward to synthesize in large amounts, induce strong chirality in polyfluorene copolymer thin films. Advanced chirality‐sensitive microscopy techniques and ultrafast time‐resolved spectroscopic methods based on transient circular dichroism and transient circularly polarized luminescence reveal the supramolecular origin of the chiroptical properties.

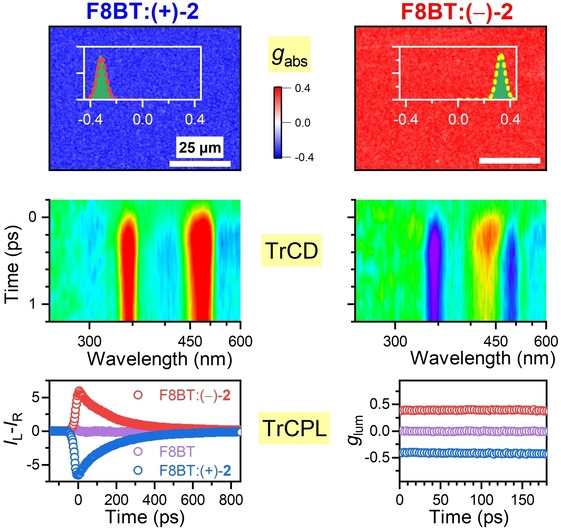

## Introduction

**Figure 1:**
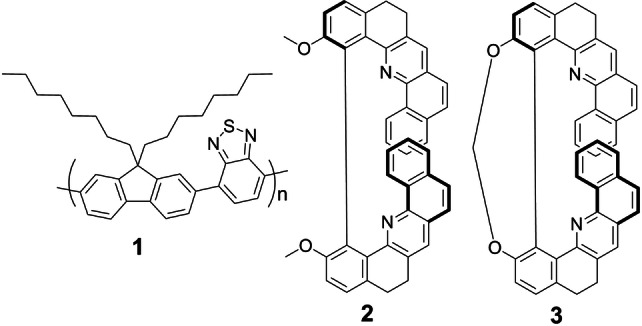
Chemical structures of the achiral F8BT copolymer **1** and the unbridged and bridged helicene‐type chiral additives **2** and **3**, respectively (shown are the (+)‐enantiomers).

Supramolecular copolymer thin films exhibiting a strong chiral response are of considerable interest for diverse optoelectronic and photonic applications, such as OLEDs with circularly polarized luminescence (CPL),[^1^, ^2^, ^3^, ^4^, ^5^] liquid‐crystal lasers,^[6]^ photodetectors,^[7]^ and polarization‐dependent security features.^[8]^ In principle, there are two approaches to obtain such chiral copolymer systems. One of them uses intrinsically chiral polymers forming supramolecular chiral structures after annealing at temperatures above the transition to the chiral nematic phase.[^9^, ^10^] Alternatively, enantiopure organic molecules can be mixed into an intrinsically achiral polymer, leading to induced chirality after annealing of the polymer‐additive blend. Regarding the latter case, helicenes have proved themselves as efficient chiral additives,[^3^, ^11^, ^12^] yet the large‐scale synthesis of enantiopure helicenes remains a difficult task.[^13^, ^14^] Recently, the Guy group succeeded in the facile preparation of enantiopure helicene‐like molecules on a gram scale.[^15^, ^16^] These molecules feature dibenzo[*c*,*h*]acridine units and exhibit quite weak CD and CPL activity. Here, we show that these helicene‐type molecules however act as highly efficient chiral additives and demonstrate this for the achiral copolymer poly‐[(9,9‐di‐*n*‐octylfluorenyl‐2,7‐diyl)‐*alt*‐(benzo[2,1,3]thiadiazol‐4,8‐diyl)], shortly F8BT (**1**). Specifically, we used 2,2′‐dimethoxy‐5,5′,6,6′‐tetrahydro‐1,1′‐bidibenzo[*c*,*h*]acridine **2** and its methylene‐bridged analog **3** (Figure 1).^[15]^ The photophysics of the pure compounds **2** and **3** has been investigated in detail previously.[^15^, ^17^]

We address open questions regarding the origin of the large induced chiral response in such films by employing steady‐state CD and CPL microscopy with diffraction‐limited resolution.^[18]^ CD microscopy also provides a clear picture of the influence of film thickness and the impact of enantiomeric excess of the chiral helicene‐type additive on the chiral properties. In addition, the dynamic response of these unique copolymer films is investigated on femtosecond time‐scales using transient broadband circular dichroism (TrCD) spectroscopy[^18^, ^19^] and a newly developed setup for transient circularly polarized luminescence (TrCPL) measurements enabling picosecond time‐resolution. Together with the results from steady‐state CD and CPL microscopy, these experiments support a supramolecular origin of the strong chirality in these systems.

## Results and Discussion

### CD and CPL Microscopy of Chiral Thin Films

Thin films were prepared by spin‐coating solutions of the copolymer **1** and the chiral additives **2** or **3** in a mixture of chlorobenzene and chloroform onto glass slides. Subsequent annealing at 150 °C provided yellow films with a thickness in the range 100–360 nm, depending on the spin‐coating conditions. Details about the film preparation process and experimental procedures are provided in the Supporting Information (Note 1).

**Figure 2:**
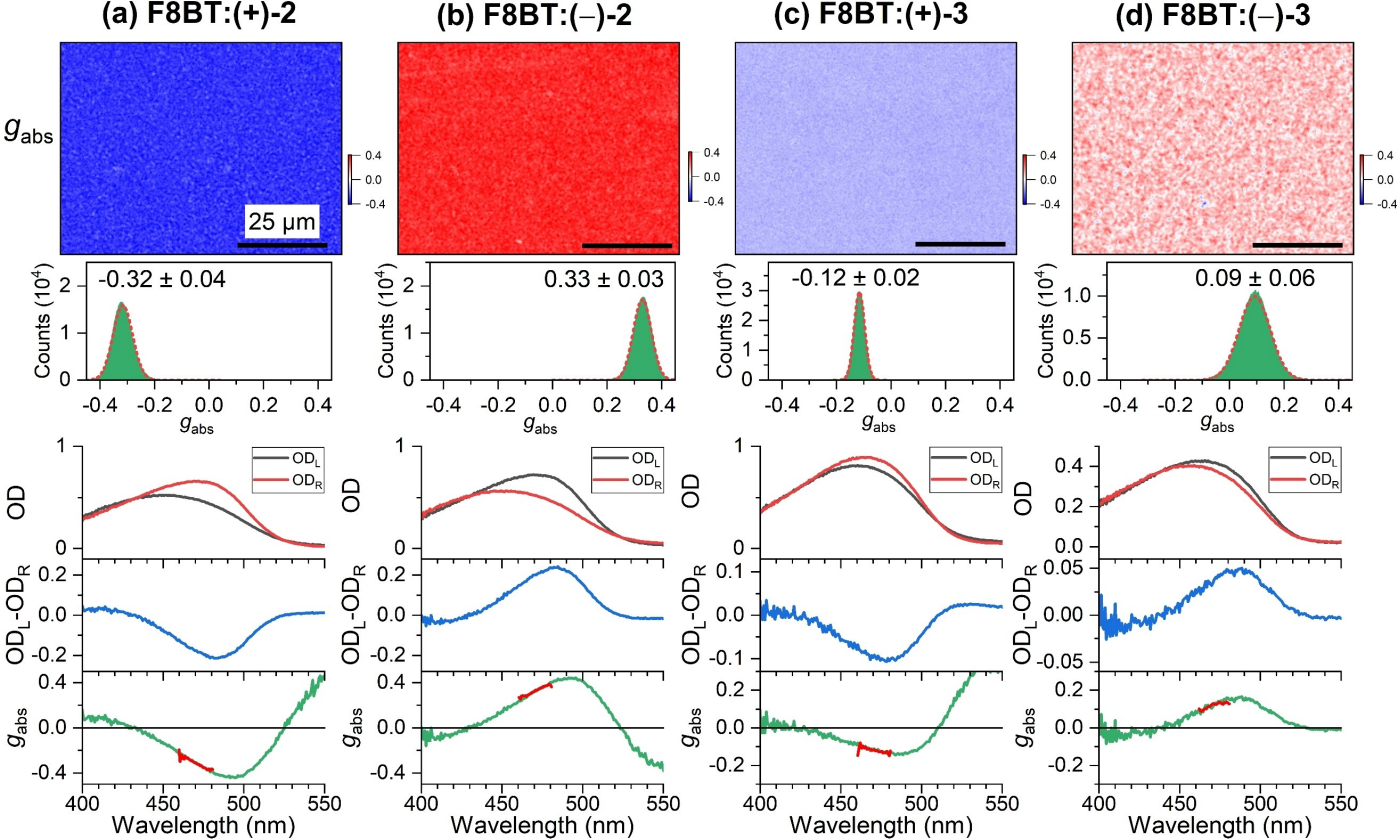
CD microscopy for blends of the achiral polymer F8BT (**1**) and the two enantiomers of the helicene‐type molecules **2** and **3**. a) Microscope image (80×60 μm^2^) showing the dissymmetry parameter *g*_abs_ of an F8BT:(+)‐**2** thin film (top) with the distribution of *g*_abs_ values (middle) including a Gaussian fit (dashed red line), determined over the entire field of view, and corresponding spectra integrated over the entire field of view (210×160 μm^2^, three panels at the bottom) displaying the optical density for left‐ and right‐circularly polarized light (OD_L_ (black line), OD_R_ (red line)), the CD spectrum (OD_L_‐OD_R_, blue line) and the *g*_abs_ spectrum (green line), with the thick red line indicating the spectral region selected by the bandpass filter (470 nm, FWHM 10 nm) used for CD imaging. b)–d) Same as in panel a, but for F8BT:(−)‐**2**, F8BT:(+)‐**3** and F8BT:(−)‐**3** thin films. The black scale bar in each CD image corresponds to a distance of 25 μm. In each case, 33 wt % of the helicene‐type additive was employed. The values for the film thickness in panels a–d were ca. 200 nm, ca. 250 nm, 202 nm and 156 nm, respectively.

The films were investigated using our very recently developed setup for CD microscopy.^[18]^ Representative data for F8BT films containing the chiral helicene‐type additives (+)‐**2**, (−)‐**2**, (+)‐**3** and (−)‐**3** are displayed in Figure 2a–d. The upper panels show CD microscope images of the dissymmetry factor *g*_abs_, which is defined as the CD signal normalized to the optical density for unpolarized light [Eq. 1]:

(1) $$g_{\mathrm{abs}}=\frac{\mathrm{CD}}{{\mathrm{OD}}_{\mathrm{unpolarized}}}=\frac{{\mathrm{OD}}_{L}-{\mathrm{OD}}_{R}}{0.5\left({\mathrm{OD}}_{L}+{\mathrm{OD}}_{R}\right)}$$

Here, OD_L_ and OD_R_ are the optical densities for the absorption of left circularly polarized (L or LCP) and right circularly polarized (R or RCP) light, respectively. Microscope images with a diffraction‐limited resolution of ca. 400 nm were recorded using a bandpass filter with a center wavelength of 470 nm (FWHM 10 nm), which is located close to the peak of the CD spectrum. As shown by the blue and red colors in Figure 2a and b, the unbridged helicene‐type chiral molecules (+)‐**2** and (−)‐**2** lead to strong chiral induction of opposite sign, reaching *g*_abs_ values of −0.32 and 0.33, respectively. The microscope images show island‐like granular structures of micrometer dimensions, consistent with a statistical orientation of the individual copolymer domains.[^1^, ^20^] Comparable textures are also observed in the crossed‐polarizer images provided in the Supporting Information (Note 2). In addition, the appearance is very similar to that of thin films formed from intrinsically chiral polyfluorene copolymers, such as c‐PFBT.^[18]^ An analysis of the complete images, containing about 175 000 data points, finds that the *g*_abs_ distributions can be well described by a Gaussian function (green areas and dashed red lines). The absorption spectra for LCP and RCP light (black and red, integrated over the complete field of view) show pronounced differences in the region of the S_0_ → S_1_ absorption band of F8BT resulting in large CD values of −0.21 and 0.24, corresponding to −7000 and 7900 mdeg, respectively (1 OD=32 982 mdeg), and peak *g*_abs_ values of −0.44 and 0.44 at 495 nm. These values rival those for helicene additives, such as 1‐aza[6]helicene.[^3^, ^11^, ^21^]

We note that annealing of the blends is absolutely required to obtain a strong chiral response, whereas pristine blends are CD‐silent (Supporting Information, Note 3). The annealed samples also did not exhibit any changes in the CD response and the dissymmetry factor *g*_abs_ upon rotation of the thin film down to the few hundred nanometer length scale. Contributions resulting from a combination of linear dichroism and linear birefringence in combination with any possible anisotropies of the CD microscopy setup can be therefore safely excluded (Supporting Information, Note 4). An additional investigation using differential scanning calorimetry (DSC) of pure F8BT and F8BT:(+)‐**2** thin films with 33 wt % of the chiral additive demonstrated that addition of (+)‐**2** barely changed the transition temperature to the chiral supramolecular phase (Supporting Information, Note 5).

Chiral induction in achiral F8BT by the bridged helicene‐type molecules (+)‐**3** and (−)‐**3** leads to qualitatively similar results (Figure 2c, d). The images show the same type of island‐like granular structures, yet, the chiral induction is weaker, as shown by the pale blue and red colors. The image analysis provides distributions, which can be well modeled by Gaussian functions with *g*_abs_=−0.12±0.02 and 0.09±0.06, respectively. The absorption spectra, integrated over the complete field of view, also show the clear but smaller difference between illumination with LCP and RCP light. We obtain peak CD values of −0.11 and 0.05, corresponding to −3500 and 1600 mdeg, respectively, with peak *g*_abs_ values at 485 nm of −0.14 and 0.16, respectively.

**Figure 3:**
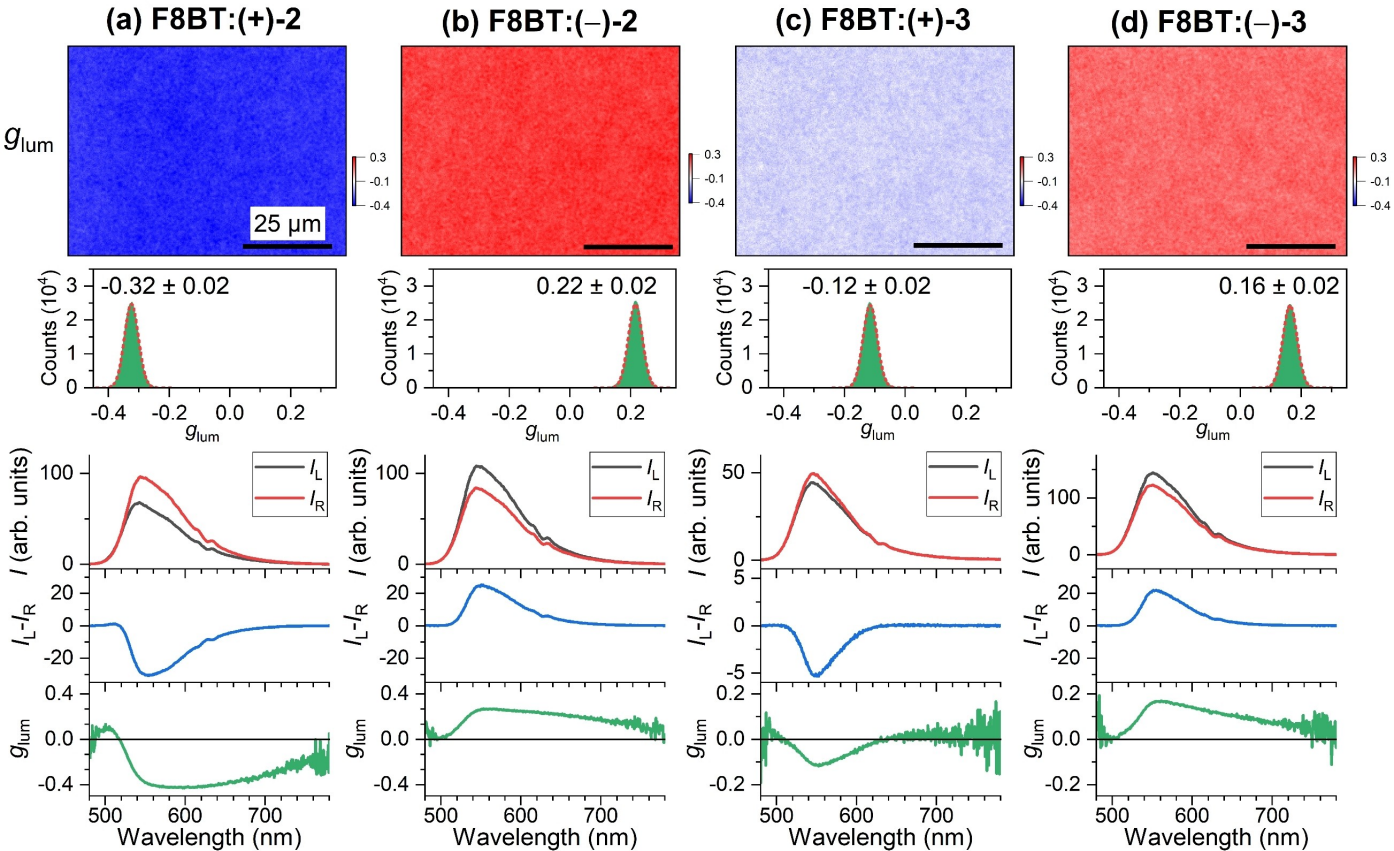
CPL microscopy for blends of the achiral polymer F8BT (**1**) and the two enantiomers of the helicene‐type molecules **2** and **3**. a) Microscope image (80×60 μm^2^) showing the dissymmetry parameter *g*_lum_ of an F8BT:(+)‐**2** thin film (top) measured using a bandpass filter with a center wavelength of 560 nm (FWHM 10 nm), with the distribution of *g*_lum_ values (middle) including a Gaussian fit (dashed red line), determined over the entire field of view, and corresponding spectra integrated over the entire field of view (210×160 μm^2^, three panels at the bottom) displaying the luminescence intensity for left‐ and right‐circularly polarized light (*I*_L_ (black line), *I*_R_ (red line)), the CPL spectrum (*I*_L_‐*I*_R_, blue line) and the *g*_lum_ spectrum (green line). b)–d) Same as in panel a, but for F8BT:(−)‐**2**, F8BT:(+)‐**3** and F8BT:(−)‐**3** thin films. The black scale bar in each CPL image corresponds to a distance of 25 μm. In each case, 33 wt % of the helicene‐type additive was employed. The values for the film thickness in panels (a)–(d) were 198 nm, 251 nm, 150 nm and ca. 150 nm, respectively.

Complementary information is provided by measuring the circularly polarized luminescence of these films using our recently introduced setup for CPL microscopy.^[18]^ Here, one can define a corresponding dissymmetry factor *g*_lum_ for CPL [Eq. 2],

(2) $$g_{\mathrm{lum}}=\frac{\mathrm{CPL}}{I_{\mathrm{unpolarized}}}=\frac{I_{L}-I_{R}}{0.5\left(I_{L}+I_{R}\right)}$$

where *I*_L_ and *I*_R_ are the luminescence intensities for LCP and RCP light, respectively. CPL images for blends of F8BT with the unbridged helicene‐type chiral molecules (+)‐**2** and (−)‐**2** are provided in Figure 3a and b. A strong chiral induction of opposite sign is clearly indicated by the blue and red colors, and the image analysis provides distributions with *g*_lum_=−0.32±0.02 and 0.22±0.02, respectively. The difference of 30 % in the *g*_lum_ values could arise from slightly different annealing temperatures, as previously observed by Wade et al.^[12]^ The structures in the CPL images have a very similar island‐like granular appearance as the CD images, but are more blurred, most likely due to reflection and scattering of the isotropic luminescence from polymer domain boundaries as well as the influence of minuscule vibrations of the microscopy setup during the fluorescence integration time of several seconds.

The corresponding luminescence spectra of the F8BT:**2** blends for LCP and RCP illumination (black and red lines, integrated over the complete field of view) show pronounced differences in intensity for the region of the S_1_ → S_0_ emission band of F8BT. This is reflected in the strong CPL spectra of opposite sign. We obtain peak *g*_lum_ values at 570 nm of −0.42 and 0.27, respectively.

CPL microscopy images for the F8BT:**3** blends are presented in Figure 3c and d. The analysis of the pale blue and pale red images provides distributions for the dissymmetry factor with *g*_lum_=−0.12±0.02 and 0.16±0.02, respectively. Differences in the LCP and RCP emission and the CPL spectra are clearly smaller than for the F8BT:**2** blends (black, red and blue lines). Peak *g*_lum_ values at 560 nm are −0.11 and 0.16. We finally would like to emphasize that there are no contributions of the chiral additives to the spectral features in Figures 2 and 3, because their absorption, emission, CD and CPL spectra are located at shorter wavelengths, as independently determined in our previous study of pure (+)‐**2**, (−)‐**2**, (+)‐**3** and (−)‐**3** thin films. The *g*_abs_ and *g*_lum_ values of these compounds both as molecules in solution and as thin films are quite small (about 10^−2^ or below). Films of the pure additive therefore do not support chiral supramolecular structures.[^15^, ^17^]

### Ultrafast Transient CD Spectroscopy and Microscopy of F8BT Blends with the Helicene‐Type Additives (+)‐2 and (−)‐2

In TrCD spectroscopy, an ultrashort laser pulse (*λ*=320 nm) promotes chiral molecules to an electronically excited state, and subsequently the sample is probed alternately by LCP and RCP broadband laser pulses covering the UV/Vis range (260–600 nm). The TrCD signal is thus obtained as [Eq. 3],

(3) $$\Delta \mathrm{CD}(t)=\Delta \Delta \mathrm{OD}(t)=\Delta {\mathrm{OD}}_{L}(t)-\Delta {\mathrm{OD}}_{R}(t)\;\;\;\;\;\;\;\;\;\;={\mathrm{OD}}_{L,\mathrm{wp}}-{\mathrm{OD}}_{L,\mathrm{wop}}-{\mathrm{OD}}_{R,\mathrm{wp}}+{\mathrm{OD}}_{R,\mathrm{wop}}$$

where “wp” means with pump beam and “wop” without pump beam. The experiment simultaneously provides information regarding the transient circular dichroism and transient absorption of the sample with a time resolution of about 100 fs. The transient absorption signal for unpolarized probing is obtained as [Eq. 4].

(4) $$\Delta {\mathrm{OD}}_{\mathrm{unpolarized}}(t)=0.5\left(\Delta {\mathrm{OD}}_{L}(t)+\Delta {\mathrm{OD}}_{R}(t)\right)$$

**Figure 4:**
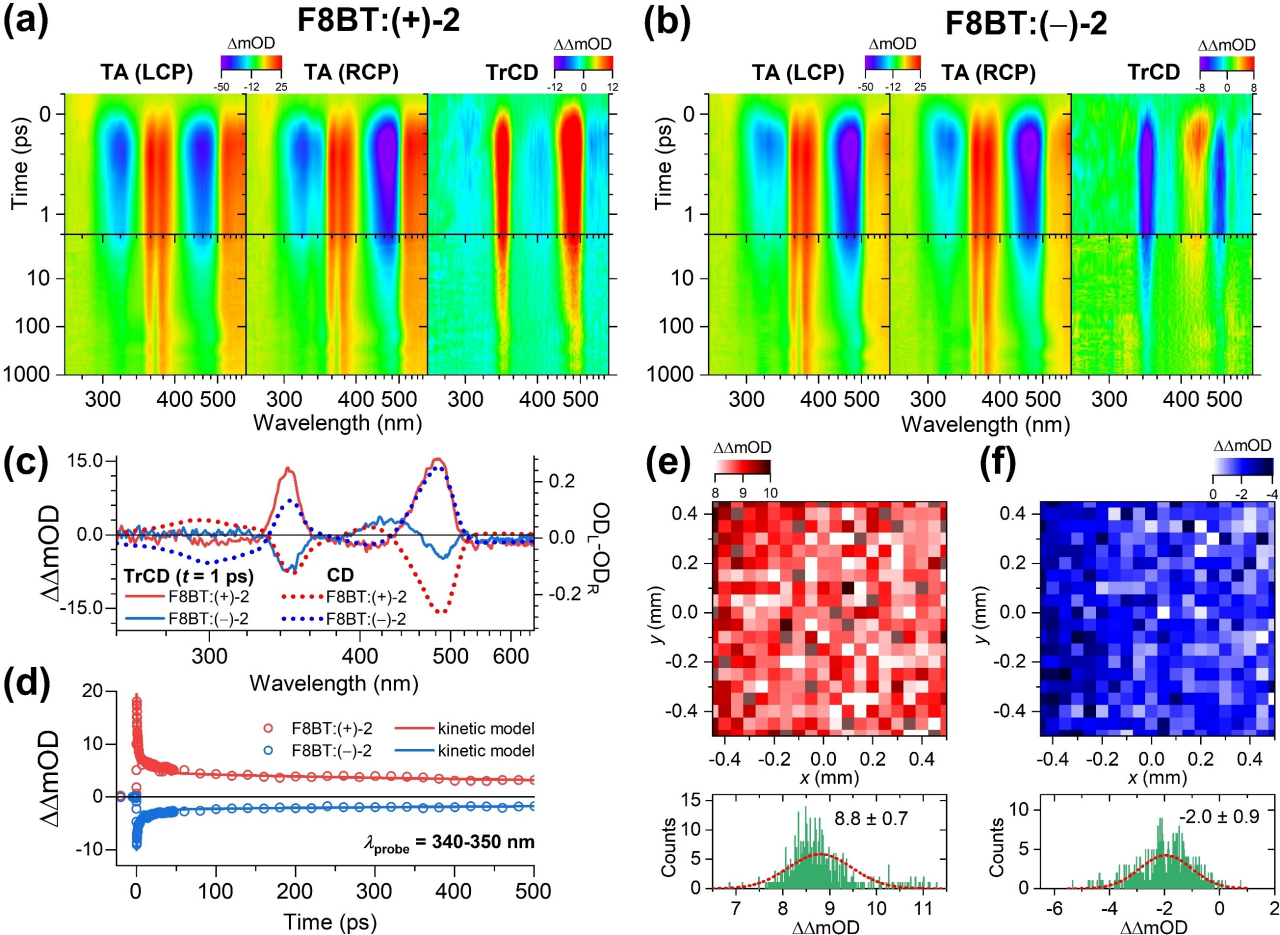
Ultrafast transient absorption and transient circular dichroism spectroscopy and microscopy of F8BT:(+)‐**2** and F8BT:(−)‐**2** thin films. a) Left: Transient absorption of F8BT:(+)‐**2** for probing with left‐circularly polarized light, middle: Transient absorption for probing with right‐circularly polarized light, right: Transient circular dichroism (difference of the contour diagrams for TA (LCP) and TA (RCP)). b) Same as in a, but for F8BT:(−)‐**2**. c) Comparison of TrCD spectra at 1 ps (solid lines) and steady‐state CD spectra (dotted lines) for F8BT:(+)‐**2** (red) and F8BT:(−)‐**2** (blue). d) Experimentally measured kinetics (open circles) averaged over the probe wavelength range 340–350 nm including fits from a kinetic model (solid lines) for F8BT:(+)‐**2** (red) and F8BT:(−)‐**2** (blue). e) Top: Ultrafast transient circular dichroism map of F8BT:(+)‐**2** at 1 ps (average: 425–525 nm). Bottom: Histogram of TrCD values from the transient CD map (green) including a Gaussian fit (red dashed line). f) Same as in (e), but for F8BT:(−)‐**2**. Note the logarithmic time axis in the contour plots of panels (a) and (b).

Details of the TrCD setup are provided in the Supporting Information (Note 1). In the following, we focus on the TrCD results for F8BT blends with the helicene‐type molecules (+)‐**2** and (−)‐**2** because of their superior chirality‐inducing properties. In the contour plot for F8BT:(+)‐**2** (Figure 4a), the transient absorption spectra for LCP and RCP detection exhibit distinct blue‐violet colored ground state bleach (GSB) features at 320 and 465 nm and pronounced yellow‐orange excited state absorption bands at 380 and 550 nm, which clearly belong to F8BT.^[22]^ We note that the GSB feature for RCP detection at 465 nm is much stronger than for LCP detection, and there is also an additional blueish sideband at 340 nm, only observed for RCP detection. The contour plot for the TrCD measurement, which corresponds to the difference of the contour plots for LCP and RCP detection, therefore, shows two positive CD features at 345 and 480 nm (orange) which must belong to the supramolecular F8BT assembly. They coincide with the position of the main peaks of the steady‐state CD spectrum of the chiral F8BT blend. The TrCD spectrum therefore looks like an inverted steady‐state CD spectrum, without indications for excited‐state CD activity (cf. Figure 4c). Its absence is consistent with a supramolecular origin of the CD signal: For a fractional excitation of about 10 % F8BT units (given by the ratio of the transient absorption bleach and the steady‐state absorbance of the sample), the excited‐state polymer species are too far apart to establish any efficient in‐plane or across‐planes couplings between the excited‐state chromophores.^[18]^ What is therefore observed is just a bleach of the ground‐state CD spectrum of the F8BT film.

If the picture above is correct, we should observe a mirror‐image like behavior in the TrCD spectra of F8BT:(−)‐**2**. This is indeed the case, as demonstrated by the contour plot of Figure 4b. Now, the GSB feature for LCP detection at 465 nm is more extended than for RCP detection, and there is a broader blueish sideband at 340 nm for LCP detection. As a result, the TrCD spectrum has negative features at 345 and 480 nm, at the same position, but of opposite sign than for F8BT:(+)‐**2** (cf. the TrCD spectra at 1 ps shown in Figure 4c). Transient CD spectroscopy is therefore able to distinguish between the two different chiral supramolecular assemblies on ultrafast time scales.

Representative kinetic traces averaged over the low‐wavelength band of the TrCD bleach region (340–350 nm) are shown for F8BT:(+)‐**2** and F8BT:(−)‐**2** in Figure 4d. Both signals exhibit a fast decay over the first few picoseconds, which is governed by singlet‐singlet annihilation. The residual signal (15 % at 1000 ps) is due to a long‐lived charge‐pair state. Detailed results from a modeling of the TrCD kinetics are provided in the Supporting Information (Note 6).

The TrCD setup also enables spatial point‐by‐point mapping of the thin films, with the 50 μm spatial resolution of the TrCD probe beam. This is demonstrated for the F8BT:(+)‐**2** and F8BT:(−)‐**2** thin films in panels e and f of Figure 4, respectively. The maps are recorded for a delay time of 1 ps and are averaged over the spectral region 425–525 nm. Again, there is a clear chiral discrimination of the two samples, with positive ΔΔOD values in the case of F8BT:(+)‐**2** (red areas), and negative ΔΔOD in the case of F8BT:(−)‐**2** (blue areas). From the statistics of the map (20×20=400 points) we obtain ΔCD values of 8.8±0.7 and −2.0±0.9 mOD from a Gaussian fit to the histograms, as shown in the lower half of panels e and f.

### Transient Circularly Polarized Luminescence Spectroscopy with Picosecond Time Resolution

Chirality‐sensitive ultrafast spectroscopic methods are valuable tools for monitoring transient electronic and structural changes of optically active molecules and supramolecular structures. Beside ultrafast transient CD spectroscopy, time‐resolved CPL approaches have been frequently utilized, as summarized very recently by Meskers.^[23]^ These include high‐sensitivity setups employing differential photon counting techniques, which are however limited to slower processes in the time range 0.01–100 ms.[^24^, ^25^, ^26^] TCSPC‐based approaches so far have reached sub‐nanosecond time‐resolution with a fairly coarse point‐by‐point spacing of about 200 ps.^[27]^

Here, we present results from a new instrument for transient CPL spectroscopy based on TCSPC with picosecond time‐resolution. We employed excitation at 410 nm (S_0_ → S_1_ transition of F8BT) by the second harmonic of an 80 MHz titanium:sapphire femtosecond laser oscillator and detection of the TrCPL signal by a fast hybrid photomultiplier detector in combination with a high‐resolution TCSPC electronics, resulting in an instrument response function of 28 ps. Single photon counting provides a very good signal‐to‐noise ratio, because high photon count rates up to 10 MHz can be harnessed with state‐of‐the‐art photon‐counting electronics and combined with extended photon accumulation times. In the current experiments for thin films with a strong chiral response, peak counts up to 2×10^5^ (channel width: 3 ps) were sufficient to arrive at a clean TrCPL signal.

**Figure 5:**
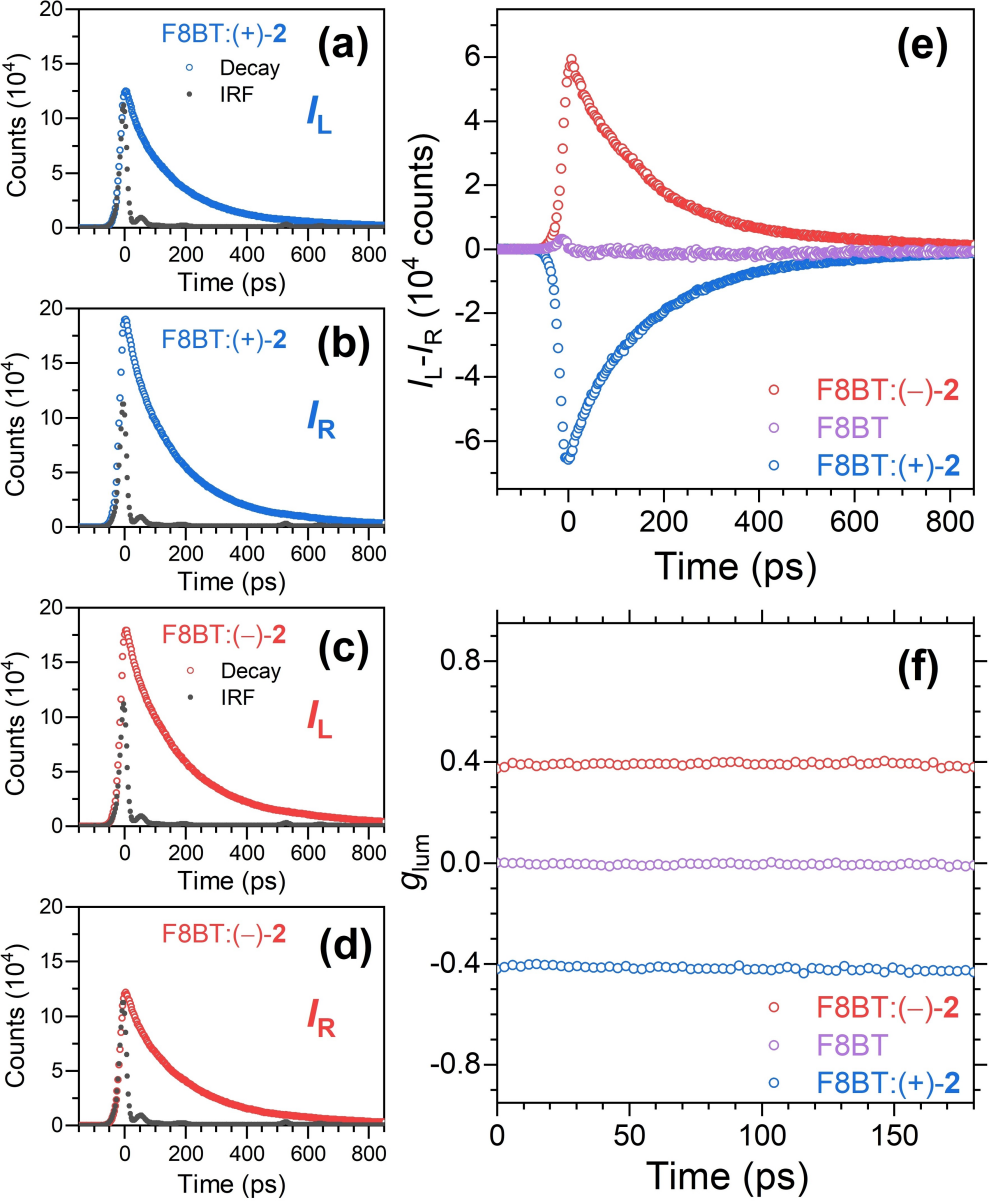
Time‐resolved CPL spectroscopy with picosecond time resolution for F8BT:(+)‐**2**, F8BT:(−)‐**2** and F8BT thin films using a bandpass filter at 530 nm. a) Transient photoluminescence intensity *I*_L_ at left circular polarization for the F8BT:(+)‐**2** film (open blue circles) and laser scattering signal *I*_L_ obtained from a colloidal SiO_2_ nanoparticle suspension (solid black circles) providing the instrument response function of the setup. b) Same as in panel a, but showing the photoluminescence intensities *I*_R_ for right circular polarization. c) and d) Same as in panels a and b, but for the F8BT:(−)‐**2** thin film and the same nanoparticle suspension. e) Resulting transient CPL signal *I*_L_‐*I*_R_ for the F8BT:(+)‐**2** thin film (blue circles) and the F8BT:(−)‐**2** thin film (red circles). Also shown is the transient CPL signal of an achiral F8BT thin film (without chiral additive, violet circles). f) Resulting time resolved *g*_lum_ signal for the F8BT:(+)‐**2** (blue circles), F8BT:(−)‐**2** (red circles) and F8BT thin films (violet circles).

Figure 5 contains our results for the chiral F8BT:(+)‐**2** and F8BT:(−)‐**2** thin films in comparison with an F8BT thin film without chiral additive. A bandpass filter centered at the emission wavelength 530 nm (10 nm FWHM) was employed. Panels a and b show the decays for the F8BT:(+)‐**2** film (blue circles) for LCP and RCP emission, respectively. The solid black circles indicate the scattering signal from a dispersion of achiral silica nanoparticles, showing the sharp detector response (with a small after‐pulse) and an FWHM of 28 ps. We note that the much larger peak intensity of the *I*_R_ transient for the thin film already indicates a substantial negative transient CPL and *g*_lum_ signal. In contrast, *I*_L_ and *I*_R_ for the scattering solution are identical. Panels c and d of Figure 5 display the corresponding results for the F8BT:(−)‐**2** film (red circles), including the same scattering signal as in panels a and b (black circles). Here, the intensity of the *I*_L_ transient of the film is much larger, indicating a positive CPL and *g*_lum_.

The resulting transient CPL signals for the two thin films, with peak signals of about 60 000 counts, are depicted in panel e. The kinetics resemble mirror images. For comparison, we also include the response of the achiral F8BT thin film (violet circles), which essentially shows no CPL signal and only a small undulation around time zero. Panel f contains the resulting time‐resolved *g*_lum_ signal. For the achiral F8BT sample *g*_lum_ is zero, whereas we observe essentially time‐independent values of −0.41 and 0.37 in the case of the chiral F8BT:(+)‐**2** and F8BT:(−)‐**2** thin films, respectively. We find very similar results in complementary TrCPL experiments using excitation at 273 nm by a UV‐LED (Supporting Information, Note 7).

The result is consistent with a supramolecular origin of CPL. The strong CPL signal likely arises from circular intensity differential scattering[^1^, ^28^] or from a mechanism where initially linearly polarized luminescence emitted from a quasi‐nematic layer is circularly polarized by passing the remaining film layer,^[29]^ or it could be a combination of both. The transient CPL signals in panel e are therefore solely due to the electronic decay of population from the S_1_ exciton state. They do not reflect any structural changes in the excited state.

### Thickness‐Dependent Chirality of F8BT Films with Helicene‐Type Additives

**Figure 6:**
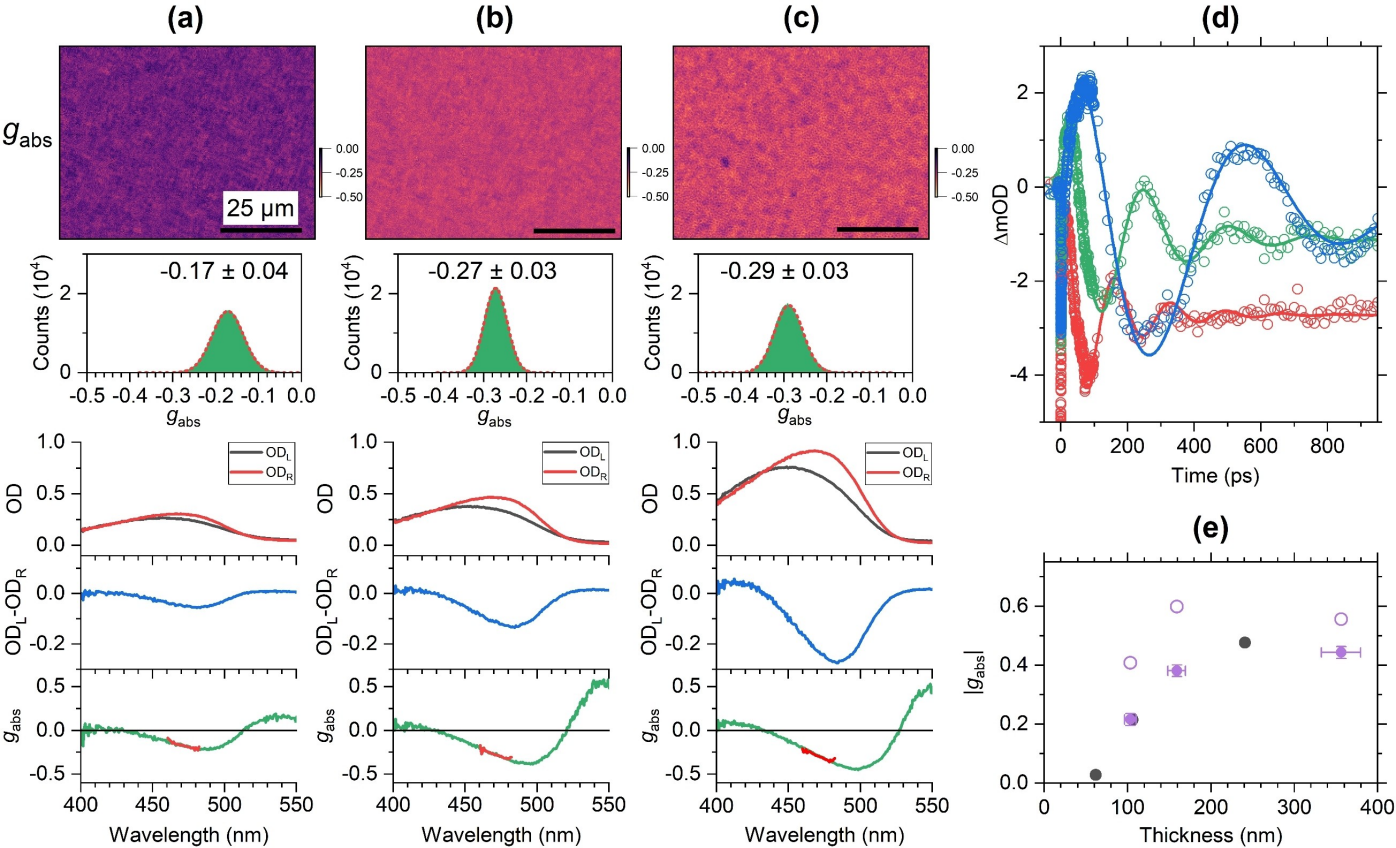
Thickness‐dependent chirality of F8BT:(+)‐**2** thin films. a) Microscope image (80×60 μm^2^) for the dissymmetry parameter *g*_abs_ of a film with the thickness *d*=103 nm (top) and a corresponding histogram of *g*_abs_ values (green) including a Gaussian fit (dashed red line), determined over the entire field of view, and spectra integrated over the entire field of view (210×160 μm^2^, bottom) showing the optical density for left‐ and right‐circularly polarized light (OD_L_ (black), OD_R_ (red)), the CD spectrum (blue) and the *g*_abs_ spectrum (green), with the thick red line indicating the spectral region selected by the bandpass filter (center wavelength 470 nm) employed for CD imaging. b) and c) Same as in panel a, but for films with the thicknesses *d*=159 and 356 nm. The length of the black scale bar in each CD image corresponds to 25 μm. d) Oscillatory transient absorption kinetics (*λ*_pump_=320 nm) averaged over the probe wavelength interval 490–520 nm (open circles) indicating the propagation of a coherent acoustic phonon in films with a thickness of 103 nm (red), 159 nm (green) and 356 nm (blue); solid lines are fits based on a sum of a biexponential term and a damped cosine term. e) absolute value of the peak of *g*_abs_ as a function of the film thickness *d*, as indicated by the violet solid circles (including error bars), open violet circles are results from the approximate reflectivity correction described in the text; corresponding *g*_abs_ values for three intrinsically chiral c‐PFBT films (data from ref. [18]) are shown as black circles for the sake of a better comparison.

Further insight regarding the origin of the very large CD signals of the F8BT:(+)‐**2** and F8BT:(−)‐**2** thin films can be gained from studying the thickness dependence of the chiral response.[^9^, ^12^, ^18^] Results for three F8BT:(+)‐**2** films of different thickness (each with 33 wt % of the chiral additive) are shown in Figure 6a–c, and additional examples are provided in the Supporting Information (Note 8). The thickness of the three films was determined in situ by picosecond ultrasonics[^18^, ^30^, ^31^, ^32^] using the transient absorption kinetics [determined via Eq. (4)] averaged over the wavelength range 490–520 nm. Characteristic oscillations appear in the transient absorption signals (Figure 6d), which arise from a coherent acoustic phonon propagating back and forth between the two interfaces of the film. The oscillation period *τ*_a_ of the acoustic phonon was obtained from a fit by a sum of a biexponential and a damped cosine term providing values of (166±1), (256±2) and (573±3) ps. The thickness *d* of the films was then determined via^[33]^ [Eq. 5],

(5) $$d=0.25\tau_{a}c_{L}$$

where *c*_L_ is the longitudinal sound velocity of the F8BT:(+)‐**2** thin film. Here, we use a value of (2490±150) m s^−1^, which we previously obtained from the closely related polyfluorene copolymer c‐PFBT.^[32]^ This way, we arrive at thicknesses of 103, 159 and 356 nm for the three films.

The microscope image of the thinnest film (panel a), recorded by a bandpass filter with the center wavelength 470 nm (FWHM 10 nm) located close to the peak of the CD spectrum, shows a granular structure. The histogram of *g*_abs_ values is well described by a Gaussian distribution with *g*_abs_=−0.17±0.04. Spectra integrated over the complete field of view, shown in the lower half of panel a, provide peak CD and *g*_abs_ values of −0.054 (−1800 mdeg) and −0.21, respectively.

Upon increasing the layer thickness to 159 nm (panel b), the average optical activity increases substantially, as indicated by the pronounced color change of the image from violet to orange, resulting in a Gaussian distribution with *g*_abs_=−0.27±0.03. The spectra provide peak values of −0.133 (−4400 mdeg) and −0.38 for the CD response and *g*_abs_, respectively. A further substantial increase of the layer thickness to 356 nm (panel c) leads to a slighter increase of *g*_abs_ (−0.29±0.03), as indicated by the brighter orange color of the image. The peak values of the CD response and *g*_abs_ are −0.276 (−9100 mdeg) and −0.44, respectively.

In the case of molecular chirality, *g*_abs_ should be independent of thickness.^[20]^ However, the correlation in Figure 6e indicates a clear increase of |*g*_abs_| up to a thickness of ca. 150 nm and a leveling‐off above (violet solid circles). This is consistent with experiments for supramolecular structures of the chiral copolymer c‐PFBT, also included in Figure 6e (black circles).^[18]^

A similar thickness dependence was found previously by Lakhwani and Meskers for thermally annealed poly‐[9,9‐bis((3*S*)‐3,7‐dimethyloctyl)‐2,7‐fluorene] films.^[20]^ This result also suggests that the large induced CD signal of these F8BT:(+)‐**2** films arises from supramolecular chirality.

We note that considering reflection losses at the interfaces will increase the values for *g*_abs_, but will not significantly change the trend in the thickness dependence of *g*_abs_. Here, we consider an approximate correction method proposed by Schulz et al.,^[34]^ which is valid for a free‐standing slab of material and will lead to an overestimation of *g*_abs_. Applying their correction, we obtain the violet open circles in Figure 6e. While all *g*_abs_ values increase, the difference Δ*g*_abs_ between the first two points remains almost the same. A short description of the model is provided in the Supporting Information (Note 9).

### Influence of the Amount of the Chiral Additive and its Enantiomeric Excess

Additional CD experiments accompanied by atomic force microscopy (AFM) measurements were performed, in which the amount of the chiral additive (+)‐**2** was varied systematically (Supporting Information, Note 10). The surface morphology recorded by AFM shows aggregated structures of less than 1 μm size which may be correlated with CD‐active regions of similar size found in the CD microscope images.

In the field of induced chirality, the issue of enantiomeric excess has been frequently discussed in terms of the “Sergeant and Soldiers” principle and “Majority Rules” concepts.[^35^, ^36^, ^37^, ^38^, ^39^] For instance, previous studies dealing with the aggregation of intrinsically chiral polythiophenes into a supramolecular structure in the poor solvent *n*‐decanol found a nonlinear relation between the *g*_abs_ value and the percent enantiomeric excess. Already for 20 % monomer enantiomeric excess, about 90 % of the maximum *g*_abs_ value was reached.^[37]^ Similar results were reported for chiral polyisocyanates.[^35^, ^36^] In such systems, chiral amplification is induced by a small enantiomeric excess of “chiral sergeants” who control the (supramolecular) chirality of a large amount of “achiral soldiers”. Here, we investigate if these concepts are still valid when mixtures of the helicene‐type additives (+)‐**2** and (−)‐**2** with different percent enantiomeric excess are present in the F8BT thin film.

**Figure 7:**
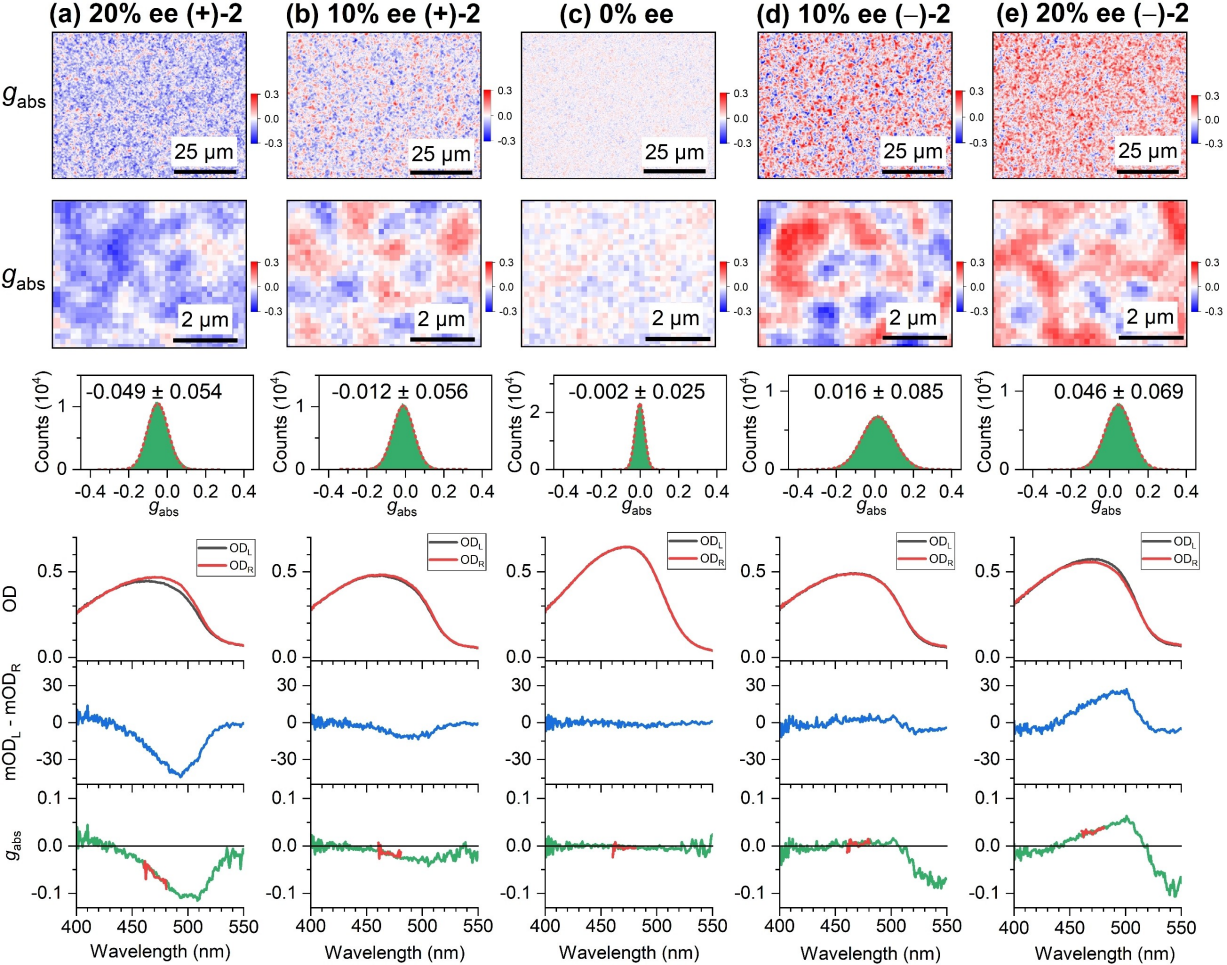
Chirality of F8BT thin films with different percent enantiomeric excess of the helicene‐type compounds (+)‐**2** and (−)‐**2**. a) Microscope image (80×60 μm^2^) including a zoom‐in of a representative region for the dissymmetry parameter *g*_abs_ of a film with 20 % ee of (+)‐**2** (top) with a histogram of *g*_abs_ values (green) including a Gaussian fit (dashed red line), determined over the entire field of view, and spectra integrated over the entire field of view (210×160 μm^2^, bottom) showing the optical density for left‐ and right‐circularly polarized light (OD_L_ (black), OD_R_ (red)), the CD spectrum (blue) and the *g*_abs_ spectrum (green), with the thick red line indicating the spectral region selected by the bandpass filter (center wavelength 470 nm) employed for CD imaging. b)–e) Same as in panel a, but for films with 10 % ee of (+)‐**2**, 0 % ee (racemic), 10 % ee of (−)‐**2** and 20 % ee of (−)‐**2**. The length of the black scale bars in the CD images corresponds to 25 μm or 2 μm. The values for the film thickness in panels (a)–(e) were 221 nm, 236 nm, 316 nm, 234 nm and 275 nm, respectively.

Figure 7 shows the results of such a systematic study. A total of 33 wt % was kept for the sum of the two enantiomers, which is the same as for the optimized conditions using only a single enantiomer, such as in Figure 2. The enantiomeric excess was varied systematically between 20 % ee of (+)‐**2** and 20 % ee of (−)‐**2**, as indicated in panels a–e. We focus first on the spectra integrated over the whole field of view shown in the lower half of each panel. Even for the extreme cases with 20 % ee of (+)‐**2** and (−)‐**2**, only weak chiral induction is observed, with absolute peak values of 0.04 (1300 mdeg) for the CD signal and 0.10 for *g*_abs_. For 10 % ee, this drops sharply to 0.01 (300 mdeg) and 0.03 for the CD and *g*_abs_ values, respectively. As expected, the racemic mixture shows no CD response. These findings are clearly not compatible with the “Sergeant and Soldiers” and “Majority Rules” principles, which would predict substantial preferred chiral induction already at 20 % ee.

Next, we focus on the *g*_abs_ images at the top of panels a–e. Here, we observe granular structures similar to those in Figure 2, however now with domains of roughly 600–900 nm diameter with positive and negative *g*_abs_ side‐by‐side, either equally distributed (racemic mixture) or dominated by the chiral additive in excess (20 % ee). It therefore appears as if there is a homogeneous distribution of regions with different *g*_abs_ over small length scales according to the individual contributions of the respective chiral additives rather than a “The winner takes it all” situation as in the “Sergeant and Soldiers” and “Majority Rules” concepts.

## Conclusion

The current investigation clearly demonstrates that helicene‐type additives based on the dibenzo[*c*,*h*]acridine motif indeed act as very efficient chiral additives for polyfluorene copolymers, such as F8BT (**1**). Interestingly, the unbridged chiral additive **2** exhibits significantly better chiral induction properties than additive **3**, which structurally differs only by one additional methylene bridge. Perhaps the less rigid structure of **2** is responsible for its better adaptation to the F8BT environment during the formation of the supramolecular chiral phase.

The supramolecular origin of the large chiral optical response, detected in absorption and luminescence, both steady‐state and time‐resolved, is underpinned by several experimental findings. The nonlinear increase of the dissymmetry parameter *g*_abs_ with film thickness is not compatible with a molecular picture of chirality, which would predict *g*_abs_ values which are independent of thickness. Furthermore, the ultrafast CD experiments only find transient spectral features of the copolymer's electronic ground state and no clear indications for transient excited‐state chirality. The latter is probably due to the weak coupling between the widely spaced excited‐state chromophores in the film, again pointing toward a supramolecular origin of the transient CD signal. Furthermore, measurements using a newly developed setup for picosecond transient CPL spectroscopy reveal a time‐independent dissymmetry parameter *g*_lum_, which is compatible with a static mechanism for the generation of CPL: Circular polarization could be imprinted on the initially emitted photons by either circular selective scattering or a change in polarization upon passing the residual supramolecular chiral film structure. Taken together, all of these findings are also compatible with the picture emerging for supramolecular chiral assemblies consisting of intrinsically chiral polymer chains.^[18]^

In the course of the current investigations it has also become more and more apparent that CD and CPL microscopy techniques with diffraction‐limited resolution are indispensable tools for understanding the chiral organization of such supramolecular assemblies. This is, for instance, underlined by the experiments dealing with the impact of the enantiomeric excess of the chiral additive on the chiral response. These measurements do not find clear indications for a “Sergeants and Soldiers” concept or “Majority Rules” underlying chiral structure formation in polyfluorene copolymer thin films. Likewise, TrCPL spectroscopy with picosecond time resolution, as presented in this study, is a welcome addition to the chiroptical toolbox and has considerable potential for the investigation of systems with much weaker chirality.

## Conflict of interest

The authors declare no conflict of interest.

1

## Supporting information

As a service to our authors and readers, this journal provides supporting information supplied by the authors. Such materials are peer reviewed and may be re‐organized for online delivery, but are not copy‐edited or typeset. Technical support issues arising from supporting information (other than missing files) should be addressed to the authors.

Supporting Information

## Data Availability

The data that support the findings of this study are available from the corresponding authors upon reasonable request.

## References

[1] D. Di Nuzzo , C. Kulkarni , B. Zhao , E. Smolinsky , F. Tassinari , S. C. J. Meskers , R. Naaman , E. W. Meijer , R. H. Friend , ACS Nano 2017, 11, 12713–12722. 29182859 10.1021/acsnano.7b07390

[2] K. Yavari , W. Delaunay , N. De Rycke , T. Reynaldo , P. Aillard , M. Srebro-Hooper , V. Y. Chang , G. Muller , D. Tondelier , B. Geffroy , A. Voituriez , A. Marinetti , M. Hissler , J. Crassous , Chem. Eur. J. 2019, 25, 5303–5310. 30714652 10.1002/chem.201806140 PMC7063663

[3] L. Wan , J. Wade , F. Salerno , O. Arteaga , B. Laidlaw , X. Wang , T. Penfold , M. J. Fuchter , A. J. Campbell , ACS Nano 2019, 13, 8099–8105. 31241299 10.1021/acsnano.9b02940

[4] L. Wan , J. Wade , X. Shi , S. Xu , M. J. Fuchter , A. J. Campbell , ACS Appl. Mater. Interfaces 2020, 12, 39471–39478. 32805911 10.1021/acsami.0c09139

[5] G. Albano , G. Pescitelli , L. Di Bari , Chem. Rev. 2020, 120, 10145–10243. 32892619 10.1021/acs.chemrev.0c00195

[6] J. Xiang , A. Varanytsia , F. Minkowski , D. A. Paterson , J. M. D. Storey , C. T. Imrie , O. D. Lavrentovich , P. Palffy-Muhoray , Proc. Natl. Acad. Sci. USA 2016, 113, 12925–12928. 27807135 10.1073/pnas.1612212113 PMC5135330

[7] M. D. Ward , J. Wade , X. Shi , J. Nelson , A. J. Campbell , M. J. Fuchter , Adv. Opt. Mater. 2021, 9, 2101044.

[8] A. J. J. Kragt , D. C. Hoekstra , S. Stallinga , D. J. Broer , A. P. H. J. Schenning , Adv. Mater. 2019, 31, 1903120. 10.1002/adma.201903120 31243825

[9] R. Abbel , A. P. H. J. Schenning , E. W. Meijer , Macromolecules 2008, 41, 7497–7504.

[10] M. J. Cho , J.-S. Ahn , Y.-U. Kim , H. A. Um , P. N. Prasad , G. J. Lee , D. H. Choi , RSC Adv. 2016, 6, 23879–23886.

[11] Y. Yang , R. Correa da Costa , D.-M. Smilgies , A. J. Campbell , M. J. Fuchter , Adv. Mater. 2013, 25, 2624–2628. 23554220 10.1002/adma.201204961 PMC3659407

[12] J. Wade , J. N. Hilfiker , J. R. Brandt , L. Liirò-Peluso , L. Wan , X. Shi , F. Salerno , S. T. J. Ryan , S. Schöche , O. Arteaga , T. Jávorfi , G. Siligardi , C. Wang , D. B. Amabilino , P. H. Beton , A. J. Campbell , M. J. Fuchter , Nat. Commun. 2020, 11, 6137. 33262352 10.1038/s41467-020-19951-y PMC7708482

[13] Y. Shen , C.-F. Chen , Chem. Rev. 2012, 112, 1463–1535. 22017405 10.1021/cr200087r

[14] B. Milde , M. Leibeling , M. Pawliczek , J. Grunenberg , P. G. Jones , D. B. Werz , Angew. Chem. Int. Ed. 2015, 54, 1331–1335; 10.1002/anie.201408637 25530612

[15] L. Guy , M. Mosser , D. Pitrat , J.-C. Mulatier , M. Kukułka , M. Srebro-Hooper , E. Jeanneau , A. Bensalah-Ledoux , B. Baguenard , S. Guy , J. Org. Chem. 2019, 84, 10870–10876. 31397566 10.1021/acs.joc.9b01465

[16] A. Bensalah-Ledoux , D. Pitrat , T. Reynaldo , M. Srebro-Hooper , B. Moore II , J. Autschbach , J. Crassous , S. Guy , L. Guy , Chem. Eur. J. 2016, 22, 3333–3346. 26797752 10.1002/chem.201504174

[17] M. Morgenroth , M. Scholz , L. Guy , K. Oum , T. Lenzer , Mol. Phys. 2021, 119, e1959072.

[18] M. Morgenroth , M. Scholz , M. J. Cho , D. H. Choi , K. Oum , T. Lenzer , Nat. Commun. 2022, 13, 210. 35017508 10.1038/s41467-021-27886-1 PMC8752614

[19] M. Scholz , M. Morgenroth , M. J. Cho , D. H. Choi , K. Oum , T. Lenzer , J. Phys. Chem. Lett. 2019, 10, 5160–5166. 31436421 10.1021/acs.jpclett.9b02061

[20] G. Lakhwani , S. C. J. Meskers , J. Phys. Chem. A 2012, 116, 1121–1128. 22148235 10.1021/jp209893h

[21] L. Wan , X. Shi , J. Wade , A. J. Campbell , M. J. Fuchter , Adv. Opt. Mater. 2021, 9, 2100066.

[22] M. Morgenroth , M. Scholz , T. Lenzer , K. Oum , J. Phys. Chem. C 2020, 124, 10192–10200.

[23] S. C. J. Meskers , ChemPhotoChem 2022, 6, e202100154.

[24] P. M. L. Blok , P. Schakel , H. P. J. M. Dekkers , Meas. Sci. Technol. 1990, 1, 126–130.

[25] D. H. Metcalf , S. W. Snyder , S. Wu , G. L. Hilmes , J. P. Riehl , J. N. Demas , F. S. Richardson , J. Am. Chem. Soc. 1989, 111, 3082–3083.

[26] D. P. Glover-Fischer , D. H. Metcalf , T. A. Hopkins , V. J. Pugh , S. J. Chisdes , J. Kankare , F. S. Richardson , Inorg. Chem. 1998, 37, 3026–3033.

[27] J. A. Schauerte , B. D. Schlyer , D. G. Steel , A. Gafni , Proc. Natl. Acad. Sci. USA 1995, 92, 569–573. 7831331 10.1073/pnas.92.2.569 PMC42783

[28] A. Sharma , A. Campbell , J. Leoni , Y. T. Cheng , M. Müllner , G. Lakhwani , J. Phys. Chem. Lett. 2019, 10, 7547–7553. 31736314 10.1021/acs.jpclett.9b02993

[29] D. Katsis , A. W. Schmid , S. H. Chen , Liq. Cryst. 1999, 26, 181–185.

[30] H. T. Grahn , H. J. Maris , J. Tauc , IEEE J. Quantum Electron. 1989, 25, 2562–2569.

[31] P. Ruello , V. E. Gusev , Ultrasonics 2015, 56, 21–35. 25038958 10.1016/j.ultras.2014.06.004

[32] M. Scholz , M. Morgenroth , M. J. Cho , D. H. Choi , T. Lenzer , K. Oum , Struct. Dyn. 2019, 6, 064502. 31893213 10.1063/1.5124438 PMC6927817

[33] C. Thomsen , J. Strait , Z. Vardeny , H. J. Maris , J. Tauc , J. J. Hauser , Phys. Rev. Lett. 1984, 53, 989–992.

[34] M. Schulz , J. Zablocki , O. S. Abdullaeva , S. Brück , F. Balzer , A. Lützen , O. Arteaga , M. Schiek , Nat. Commun. 2018, 9, 2413. 29925832 10.1038/s41467-018-04811-7 PMC6010436

[35] M. M. Green , M. P. Reidy , R. J. Johnson , G. Darling , D. J. O′Leary , G. Willson , J. Am. Chem. Soc. 1989, 111, 6452–6454.

[36] M. M. Green , B. A. Garetz , B. Munoz , H. Chang , J. Am. Chem. Soc. 1995, 117, 4181–4182.

[37] B. M. W. Langeveld-Voss , R. J. M. Waterval , R. A. J. Janssen , E. W. Meijer , Macromolecules 1999, 32, 227–230.

[38] L. J. Prins , P. Timmerman , D. N. Reinhoudt , J. Am. Chem. Soc. 2001, 123, 10153–10163. 11603964 10.1021/ja010610e

[39] S. Huang , H. Yu , Q. Li , Adv. Sci. 2021, 8, 2002132. 10.1002/advs.202002132 PMC8061372 33898167

