# Supplementary material for: Spatiotemporal Mapping of Efficient Chiral Induction by Helicene‐Type Additives in Copolymer Thin Films
Source: Angew Chem Int Ed Engl. 2022 Jun 23;61(31):e202203075. doi: 10.1002/anie.202203075 (PMC9401025; doi:10.1002/anie.202203075)
Supplement: Supplementary file 1 — Supporting Information [file ANIE-61-0-s001.pdf]

Angewandte Chemie
Eine Zeitschrift der Gesellschaft Deutscher Chemiker

Supporting Information

Spatiotemporal Mapping of Efficient Chiral Induction by Helicene-Type Additives in Copolymer Thin Films

M. Morgenroth, M. Scholz, L. Guy, K. Oum, T. Lenzer**

Supporting Information

Table of Contents

Supporting Note 1. Experimental Details S2

Supporting Note 2. Crossed-Polarizer Images of F8BT:(+)-**2** Thin Films S6

Supporting Note 3. Chiral Response of Pristine and Annealed F8BT:(+)-**2** Thin Films S7

Supporting Note 4. Invariance of CD Signals of F8BT:**2** Thin Films Under Sample Rotation ... S10

Supporting Note 5. DSC Measurements for Pure F8BT and an F8BT:(+)-**2** Blend S12

Supporting Note 6. Ultrafast Electronic Relaxation of F8BT:(+)-**2** and F8BT:(–)-**2** Blends S14

Supporting Note 7. Transient CPL Measurements Employing Excitation by a UV-LED S19

Supporting Note 8. Dependence of CD Signals on Film Thickness S20

Supporting Note 9. Reflectivity Correction for the Dissymmetry Parameter g_{abs} S22

Supporting Note 10. CD and AFM Experiments for Different F8BT:(+)-**2** Blends S23

Supporting References S26

Supporting Note 1. Experimental Details

1.1 Preparation of Glass Slides for Spin-Coating

Microscope glass slides (Duran Wheaton Kimble) were treated in soapy distilled water and afterwards in acetone in an ultrasonic bath for 30 minutes each. After drying with nitrogen, the glass slides were cut into pieces to fit the chuck of the spin coater. The slides were treated in a UV chamber (Dinies ELG100S, 2 UV-C lamps with 253.7 nm emission, 11 W each) for 15 minutes.

1.2 Preparation of Thin Films

The chiral helicene compound 2,2'-dimethoxy-5,5',6,6'-tetrahydro-1,1'-bidibenzo[*c*,*h*]acridine **2** and its methylene-bridged analog **3** were prepared as described previously.[1] A mixture of poly-[(9,9-di-*n*-octylfluorenyl-2,7-diyl)-*alt*-(benzo[2,1,3]thiadiazol-4,8-diyl)], shortly F8BT or **1**, (Merck, $M_n \leq$ 25000 g mol^{-1}) and one of the enantiopure helicene-type compounds was dissolved in a solution of chlorobenzene (Merck, > 99%) and chloroform (Merck, ≥ 99.8%) in a ratio of 1:9 by volume. For some experiments, the compounds were also dissolved in toluene. The concentrations of F8BT and the chiral additive (CA) were 7.50 mg mL^{-1} and 3.75 mg mL^{-1} (i.e. $m_{CA}/(m_{CA}+m_{F8BT})$ = 33 wt% of the CA), providing the highest CD values. For systematic studies, other weight percentages were also tested. The solution was treated in an ultrasonic bath for 60 minutes and filtered through a 0.45 µm PTFE filter using a syringe. The precursor solution was then spin-coated onto the glass slides (without alignment layer) at 500 rpm for 60 s in a nitrogen atmosphere (humidity: < 1%) and subsequently annealed on a hot plate at 150 °C for 15 minutes.

1.3 Preparation of Thin Films with Different Enantiomeric Excess Ratios

Precursor solutions of the copolymer and the chiral additive (+)-**2** and (–)-**2** were prepared for different enantiomeric excess (ee) ratios using concentrations of 7.50 mg mL^{-1} for F8BT and 3.75 mg mL^{-1} for compound **2**. They were spin-coated and heat-treated as described above. Concentrated stock solutions of F8BT and the enantiopure additives were prepared in a mixture of chlorobenzene and chloroform (1:9 v:v) and treated by ultrasound for 60 minutes. The volumes required were gaged by a microliter pipette, mixed and then filtered through a 0.45 µm PTFE filter using a syringe. Values for the percent enantiomeric excess of the chiral helicenes were 20%, 10% and 0% (racemic mixture). Concentrations and volumes are summarized in Supporting Table 1.1.

Supporting Table 1.1. Concentrations and volumes for solutions of F8BT and the (+)-2 and (–)-2 enantiomers used for preparing blends with different percent enantiomeric excess of the chiral additive. A total volume of 0.4 mL precursor solution was sufficient for spin-coating two thin films.

		%ee				
		20 for (–)-**2**	10 for (–)-**2**	0 (racemic)	10 for (+)-**2**	20 for (+)-**2**
c (mg mL^{-1})	F8BT (**1**)	12	12	12	12	12
c (mg mL^{-1})	(+)-**2**	10	10	10	10	10
c (mg mL^{-1})	(–)-**2**	10	10	10	10	10
V (mL)	F8BT (**1**)	0.25	0.25	0.25	0.25	0.25
V (mL)	(+)-**2**	0.06	0.07	0.08	0.08	0.09
V (mL)	(–)-**2**	0.09	0.08	0.08	0.07	0.06
V_{total} (mL)	Mixture	0.40	0.40	0.40	0.40	0.40
c_{total} (mg mL^{-1})	F8BT (**1**)	7.50	7.50	7.50	7.50	7.50
c_{total} (mg mL^{-1})	(+)-**2**	1.50	1.69	1.88	2.06	2.25
c_{total} (mg mL^{-1})	(–)-**2**	2.25	2.06	1.88	1.69	1.50

1.4 Differential Scanning Calorimetry (DSC)

F8BT and an F8BT:(+)-**2** mixture (with 33 wt% of the chiral additive) were each dissolved in THF. The concentration of F8BT and the mixture were 12 mg mL^{-1} and the solutions were treated in an ultrasonic bath for 60 minutes. Afterwards, the solutions were filtered through 0.45 µm PTFE filters with a syringe. The solutions were dripped step-by-step into aluminum crucibles (Netzsch, 25 µL) and dried under a flow of nitrogen. The procedure was repeated until a mass of about 10 mg was reached. The pans were then further dried in a nitrogen atmosphere with less than 1% humidity for about 12 hours. Prior to the measurement, small holes were pierced into the lid, then the crucible and the lid (Supporting Figure 1.1) were pressed together and the exact mass was measured.

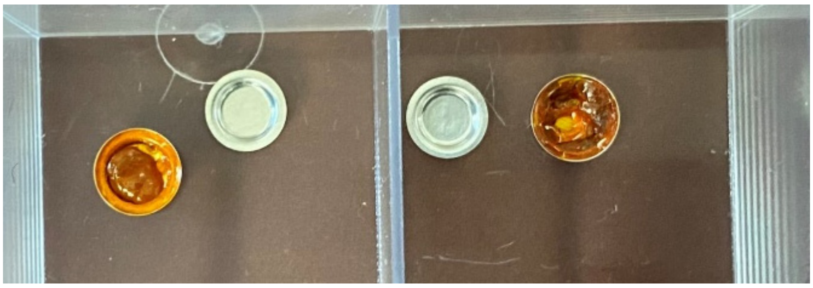

Supporting Figure 1.1. Image of the aluminum crucibles containing the samples. Left side: F8BT:(+)-**2** (33 wt% chiral additive). Right side: pure F8BT.

DSC data were collected using a thermo-microbalance-based simultaneous TG-DSC instrument (Netzsch STA 449 C Jupiter). An empty aluminum crucible served as a reference. Two independent nitrogen gas flows (20 ml min^{-1} each) and an additional argon gas flow (20 ml min^{-1}) were employed for purging. Multiple temperature segments were traversed and are summarized in Supporting Table 1.2. A fixed heating rate of 10 °C min^{-1} was used for the heating and cooling segments. The first segment went up to a temperature of 80 °C to evaporate residual THF. After cooling down to about 30 °C, the next segment went up to a temperature of 170 °C. According to Donley et al., the glass transition temperature of F8BT samples with different molecular weights (M_n = 9–255 kg mol^{-1}) is in the range 135–140 °C.[2] A temperature of 170 °C should therefore provide a well resolved signal for the glass transition and a non-destructive first scan for the F8BT:(+)-**2** blend, as we observed beginning decomposition of the chiral additive above 200 °C. After cooling down to 30 °C, two heating cycles to 320 and 330 °C, respectively, were carried out, each one followed by cooling to 30 °C.

Supporting Table 1.2. Temperature program of the DSC measurements.

Segment	$T_{initial}$ (°C)	T_{final} (°C)	Heating rate (°C min^{-1})
1	25	80	10
2	80	30	10
3	30	170	10
4	170	30	10
5	30	320	10
6	320	30	10
7	30	330	10
8	330	30	10

1.5 Atomic Force Microscopy (AFM)

Images of the topography of annealed F8BT and F8BT:(+)-**2** thin films (with 9, 17 and 33 wt% of the chiral additive) were taken with an atomic force microscope (PSIA XE-100) in non-contact mode using a cantilever with a silicon tip. The resolution was 512 times 512 pixel for an area of 6×6 µm^2, with a scan rate of 0.5 Hz. The AFM images were evaluated using the software package Gwyddion[3] (version 2.61) and visualization was carried out using OriginPro2022 (OriginLab Corporation). Forward and backward scans were averaged and afterwards background corrected.

1.6 Steady-State Absorption Spectroscopy and Microscopy

A Varian Cary 5000 spectrophotometer was employed to measure steady-state absorption spectra of macroscopic (5×5 mm^2) thin film areas using a slit width of 0.5 nm. CD spectra were recorded on the same spectrometer by adding a home-built assembly featuring polarizer – achromatic quarter-wave plate combinations covering the UV–Vis range, as described previously.[4] For systematic measurements of the chiral response for different weight ratios of the copolymer and the chiral additive, we also used an Applied Photophysics Chirascan instrument, which was kindly provided by the Ihmels group (Organic Chemistry 2, University of Siegen). Circular dichroism, circularly polarized luminescence and crossed-polarizer microscopy experiments of the thin film samples were performed on a recently established setup, which is built around a modified Olympus IX71 inverted microscope, providing images with a diffraction-limited resolution of about 400 nm as well as CD and CPL spectra integrated over the entire field of view.[4]

1.7 Ultrafast Broadband Transient Circular Dichroism and Absorption Spectroscopy

Transient circular dichroism and transient absorption spectra were recorded on a setup based on an amplified titanium:sapphire laser system (Coherent Libra USP-HE), as described previously.[4] The thin film samples, which were kept inside an aluminum cell under a constant flow of dry nitrogen, were excited at 320 nm and probed by broadband multifilament UV–Vis supercontinuum pulses (260–700 nm), which were either alternately left and right circularly polarized (TrCD experiments) or linearly polarized (transient absorption measurements). The kinetic analysis of the TrCD data was performed using the program Tenua 2.1.[5] The rate constants in the mechanism were varied to arrive at an optimal description of the TrCD kinetics.

The thickness of the polymer films was determined from the transient absorption kinetics using picosecond ultrasonics[6,7] assuming that the longitudinal sound velocity of the thin films is equal to that of the previously investigated closely related copolymer c-PFBT (υ_L = 2490 m s^{-1}).[8] Error bars for the thickness were determined from an error propagation analysis considering the known uncertainty of υ_L[8] and the uncertainty of the period of the coherent acoustic phonon oscillation obtained from the fit.

1.8 Time-Correlated Single Photon Counting

A new setup for time-correlated single photon counting (TCSPC) was constructed to record transient photoluminescence decays. The samples were photoexcited by the second harmonic (410 nm) of a mode-locked titanium:sapphire oscillator (Spectra-Physics Tsunami, pulse duration 80 fs, 80 MHz repetition frequency). Tuning of the polarization and the laser pulse energy was achieved by the combination of a Glan-Taylor polarizer (Karl Lambrecht, MGTYS10) and a tunable zero-order half-wave phase retardation plate (Alphalas, PO-TWP-L2-25-UVIR). The photoluminescence of the samples was collected at an angle of 90° by a quartz lens, sent through a bandpass filter (Thorlabs,

FB530-10, center wavelength 530 nm, FWHM 10 nm), and then refocused by another quartz lens onto a hybrid multialkali photodetector with a cathode diameter of 3 mm (Becker & Hickl, HPM-100-07). The signal pulses from the detector and the synchronization pulses of the laser (recorded by a fast photodiode, Becker & Hickl, PHD-400-N-SET) were fed into the inputs of a TCSPC module (Becker & Hickl, SPC-130IN) which was operated in reversed start-stop configuration.

For lifetime measurements, the samples were excited by vertically polarized laser light (0°) and the photoluminescence was recorded at the magic angle (54.7°) employing a wire-grid polarizer (Thorlabs WP25M-UB) in the photoluminescence detection path. For time-resolved CPL measurements, the 410 nm laser pulses were sent through a Hanle depolarizer to scramble the polarization prior to excitation. In the detection path, an achromatic broadband quarter-wave plate (Thorlabs AQWP05M-580) was inserted between the collecting lens and the polarizer. CPL kinetics were obtained by subtracting the decays of two consecutive TCSPC measurements (recorded for identical collection times) with the polarizer axis fixed at 0° and the fast axis of the quarter-wave plate set at either +45° or –45° with respect to the polarizer axis. An instrument response function (IRF) of 28 ps was determined from the laser scattering signal of a diluted suspension of colloidal silica nanoparticles (Merck, Ludox AM-40). The time constants and amplitudes of the thin-film photoluminescence decays were obtained from tail fits employing the FAST program (Edinburgh Instruments).

Additional TCSPC experiments with lower time-resolution were carried out using a pulsed UV-LED (Becker & Hickl, UVL-FB-270, 273 nm, 20 MHz repetition frequency) as the excitation source. For lifetime measurements, the output of the LED was vertically polarized (0°) by means of a wire-grid polarizer (Thorlabs WP25M-UB). For time-resolved CPL measurements, the samples were excited directly by the unpolarized output pulses of the LED. For these experiments, the time resolution was limited by the LED pulse width of 500 ps.

Supporting Note 2.
Crossed-Polarizer Images of F8BT:(+)-2 Thin Films

In Supporting Figure 2.1 we present crossed-polarizer images of different F8BT:(+)-**2** thin films. The upper row contains images of a pristine film (panel a) and an annealed (150 °C) film (panel b) spin-coated from a chlorobenzene:chloroform mixture. The lower row shows similar images, but for films spin-coated from toluene. The black images in panels a and c indicate that the pristine (as-deposited) films do not turn the initially linear polarization of the light. This is consistent with the presence of only weak molecular optical activity due to the embedded chiral helicene-type additive. In contrast, the crossed-polarizer images of the annealed films in panels b and d show characteristic granular textures, which are consistent with the structures observed in the CD and CPL images (cf. Figures 2, 3, 6 and 7 in the main manuscript). The strong optical activity of the annealed films indicates the formation of a chiral supramolecular arrangement.

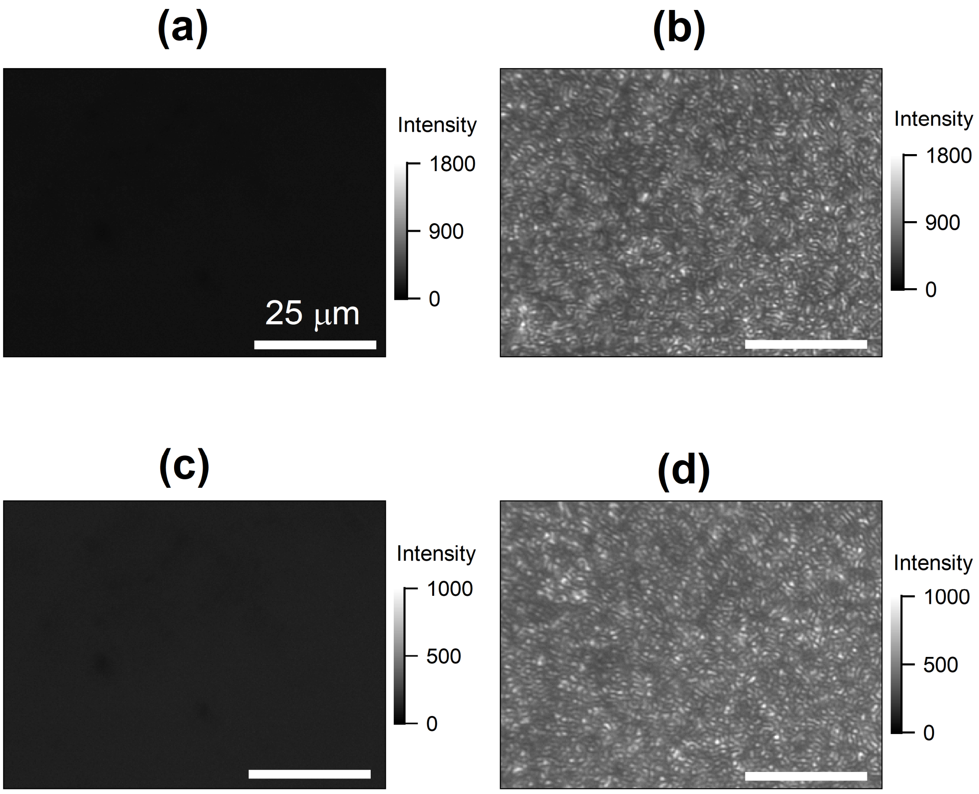

Supporting Figure 2.1. Crossed-polarizer images for F8BT:(+)-2 thin films. a) Pristine thin film spin-coated from a mixture of chlorobenzene and chloroform. b) Annealed thin film originally spin-coated from a mixture of chlorobenzene and chloroform. c) Pristine thin film spin-coated from toluene. d) Annealed thin film originally spin-coated from toluene. Blends were deposited employing 33 wt% of the chiral additive. Images for a/b and c/d were recorded for identical conditions. The white scale bar in each image corresponds to a distance of 25 μm.

Supporting Note 3.
Chiral Response of Pristine and Annealed F8BT:(+)-2 Thin Films

In Supporting Figure 3.1 we present CD microscope images and spectra of F8BT:(+)-**2** thin films spin-coated from a chlorobenzene:chloroform mixture (1:9 v:v) in pristine form (panel a) and after annealing at 150 °C (panel b). Panels c and d show corresponding data, but for these thin films the spin-coating was performed from toluene. The images and spectra of the pristine films in panels a and c show no CD response, whereas the annealed films in panels c and d exhibit pronounced CD activity.

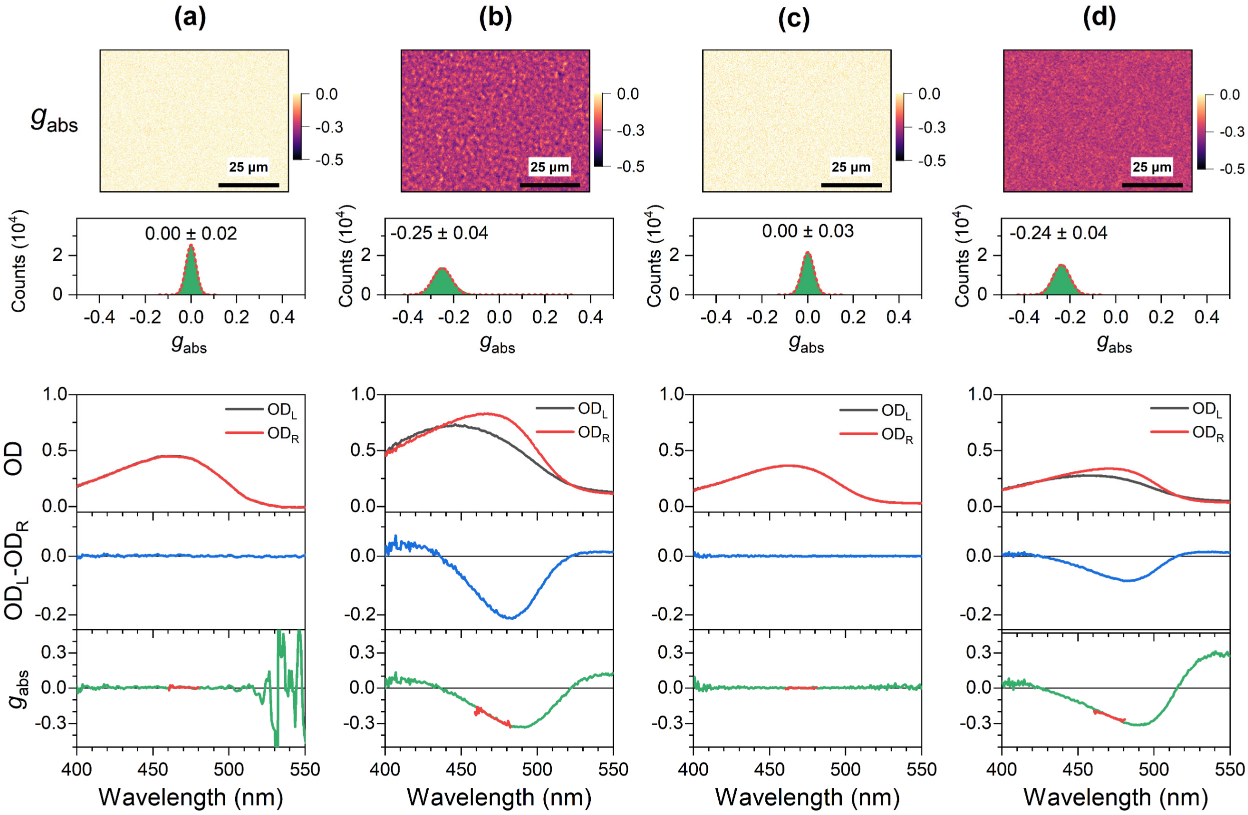

Supporting Figure 3.1. CD microscopy of pristine and annealed F8BT:(+)-2 thin films spin-coated from different solvents. a) Microscope image (80 × 60 μm^2) showing the dissymmetry parameter g_{abs} of a pristine F8BT:(+)-2 thin film spin-coated from a chlorobenzene:chloroform mixture (top) with the distribution of g_{abs} values (middle) including a Gaussian fit (dashed red line), determined over the entire field of view, and corresponding spectra integrated over the entire field of view (210 × 160 μm^2, three panels at the bottom) displaying the optical density for left- and right-circularly polarized light (OD_L (black line), OD_R (red line)), the CD spectrum (OD_L-OD_R, blue line) and the g_{abs} spectrum (green line), with the thick red line indicating the spectral region selected by the bandpass filter (470 nm, FWHM 10 nm) used for CD imaging. b-d) Same as in panel a, but for an annealed F8BT:(+)-2 thin film spin-coated from a chlorobenzene:chloroform mixture, a pristine F8BT:(+)-2 thin film spin-coated from toluene, and an annealed F8BT:(+)-2 thin film spin-coated from toluene, respectively. The scale bar in each CD image corresponds to a distance of 25 μm. In each case, 33 wt% of the helicene-type additive was employed.

Supporting Figure 3.2 shows bulk steady-state absorption spectra (top) for LCP and RCP probing (dashed and solid lines, respectively), CD spectra (middle) and g_{abs} spectra (bottom) for annealed thin films of F8BT:(+)-**2** thin films spin-coated from a chlorobenzene:chloroform mixture (1:9 v:v, black lines) or from toluene (red lines), and also for a thin film of the pure helicene-type additive (+)-**2** (blue lines). After deposition, all films were annealed at 150 °C. The F8BT:(+)-**2** blends exhibit a strong chiral response. The large CD values arise from supramolecular chirality. In contrast, on the same OD scale, the thin film of the pure helicene-type additive (+)-**2** does not show any appreciable optical activity, which suggests molecular chirality, i.e. the thin film of the chiral additive does not form a chiral supramolecular arrangement.

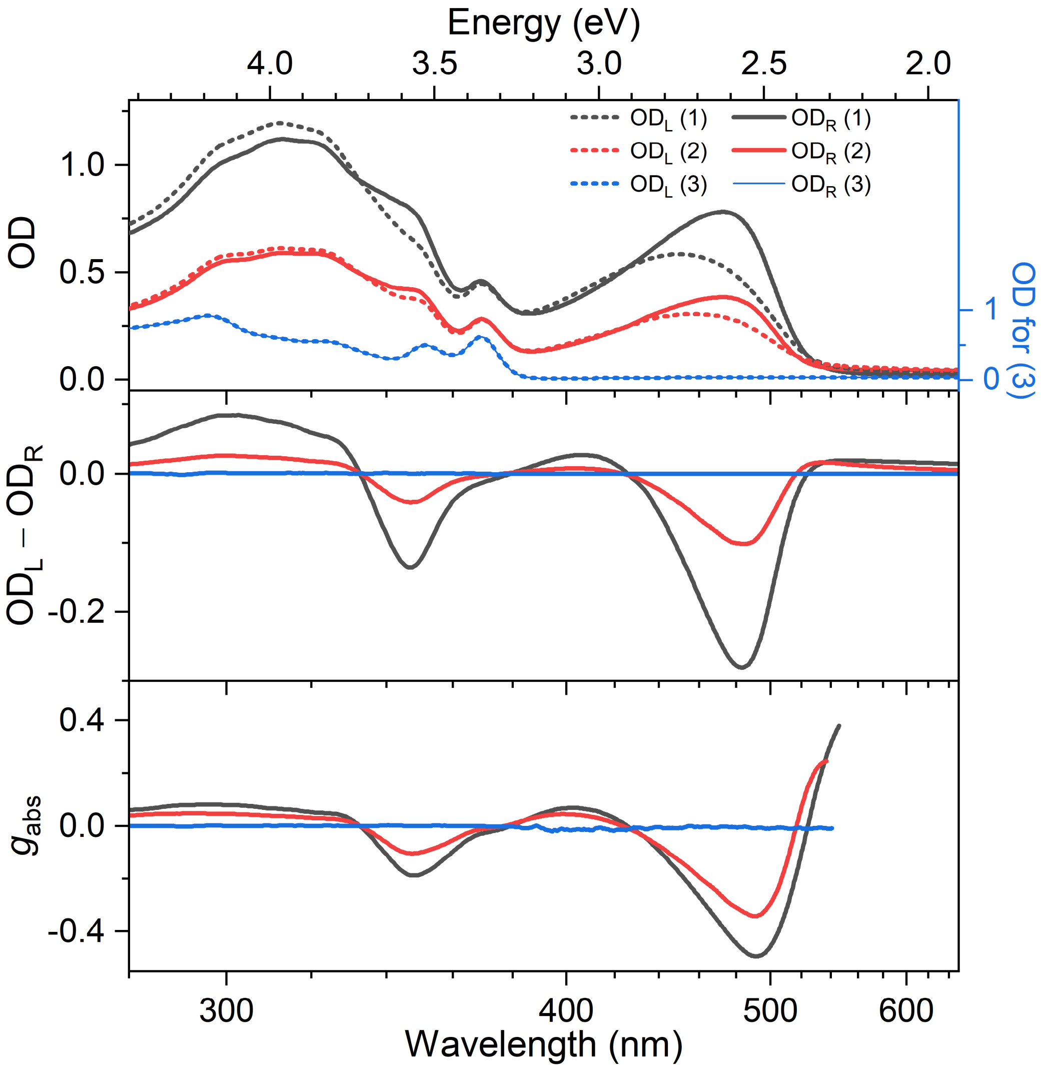

Supporting Figure 3.2. Steady-state optical spectra for annealed F8BT:(+)-2 thin films spin-coated from a chlorobenzene:chloroform mixture (black, 1) or toluene (red, 2) and for an annealed thin film of pure compound (+)-2 (blue, 3). Top) Absorption spectra for LCP (dashed lines) and RCP detection (solid lines). Middle) CD spectra. Bottom) g_{abs} spectra. Blends were deposited employing 33 wt% of the chiral additive.

In Supporting Figure 3.3, we provide spectra for the pristine (= as deposited) thin films for comparison. In this case, the thin films of the blends do not show CD activity. Formation of a chiral supramolecular arrangement therefore requires annealing of the blends above the transition temperature.

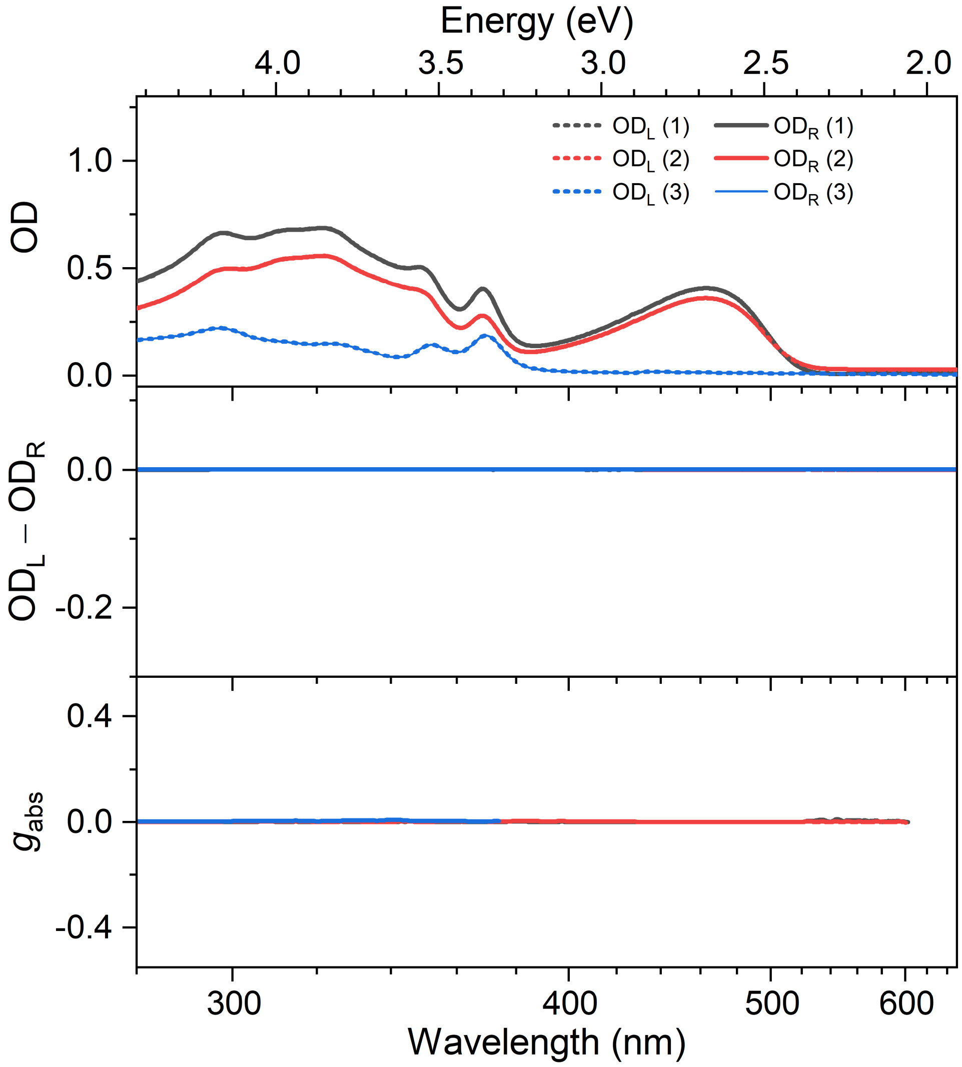

Supporting Figure 3.3. Same as in Supporting Figure 3.2, but for the pristine (as deposited) films.

Supporting Note 4.
Invariance of CD Signals of F8BT:2 Thin Films Under Sample Rotation

Supporting Figure 4.1 shows CD microscope images and spectra of an F8BT film containing 33 wt% of an enantiomer mixture of the helicene-type chiral additive **2**, with 10% enantiomeric excess of (–)-**2**. Under these conditions, small domains of opposite chirality with high g_{abs} contrast can be easily distinguished in the CD microscope, as indicated by the small red and blue regions appearing side-by-side in the overview microscope images in the top row of the figure. This property makes such films ideal candidates to check the invariance of the chiral response upon sample rotation, even for very small regions with length scales on the order of the diffraction limit.

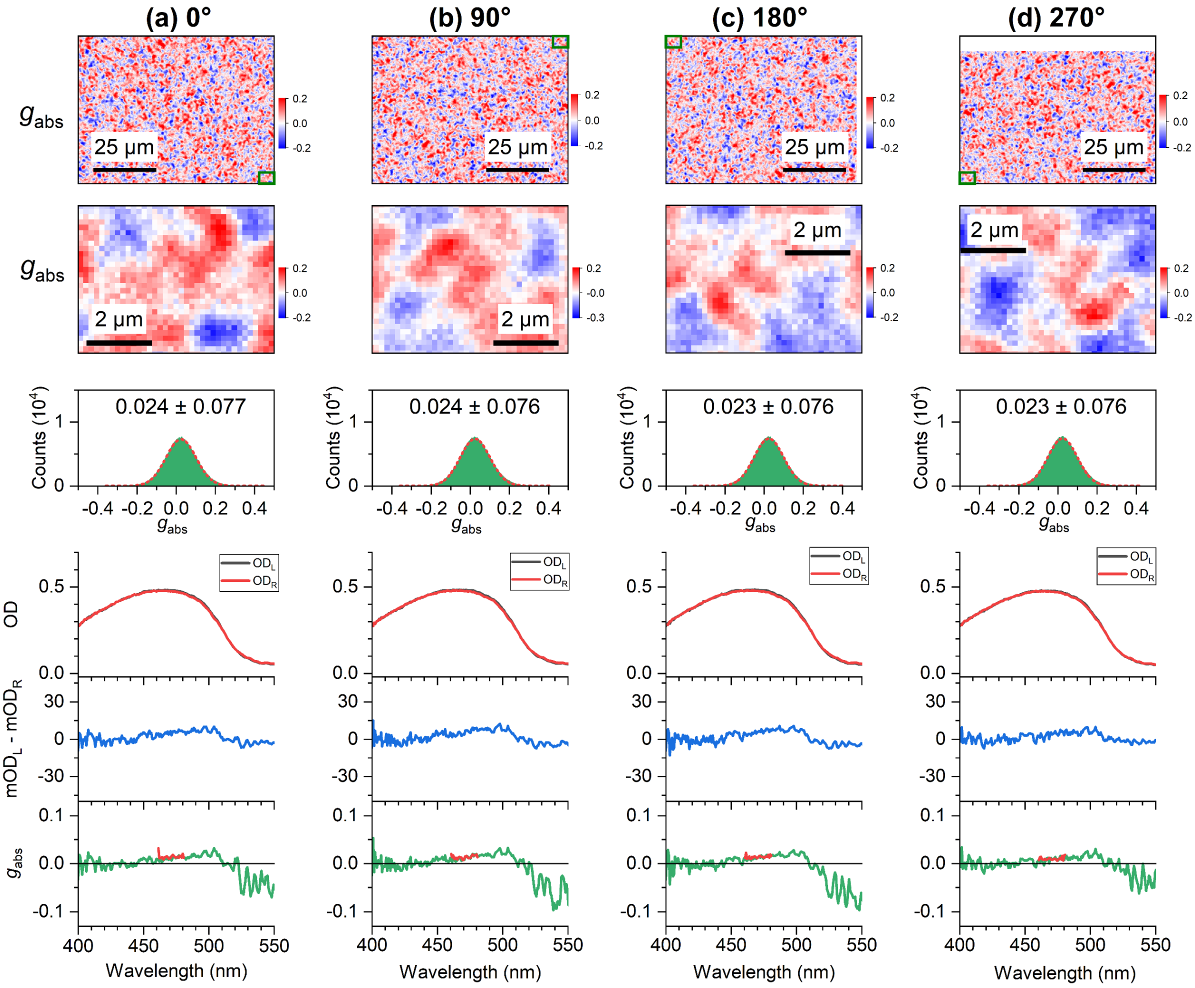

Supporting Figure 4.1. Invariance of CD microscope images under rotation for an F8BT:2 thin film with 10% enantiomeric excess of the helicene-type compound (–)-2. a) Microscope image (80 × 60 μm^2, top row) including a zoom-in of a representative region (second row, indicated by the small rectangular region in the top row) for the dissymmetry parameter g_{abs}, with a histogram of g_{abs} values (green) including a Gaussian fit (dashed red line), determined over the entire field of view (third row), and spectra (bottom row) integrated over the entire field of view (210 × 160 μm^2) showing the optical density for left- and right-circularly polarized light (OD_L (black), OD_R (red)), the CD spectrum (blue) and the g_{abs} spectrum (green), with the thick red line indicating the spectral region selected by the bandpass filter (470 nm, FWHM 10 nm) employed for CD imaging. b-d) same as in panel a, but for a rotation of the sample by 90°, 180° and 270°, respectively.

In the second row, we zoom into a specific region of the film (indicated by a rectangle in each of the images in the first row). The selected part of the image features a prominent horseshoe-shaped red region of positive g_{abs}. One camera pixel in each of these images represents a CD measurement for a 160 x 160 nm^2 area, and the estimated optical resolution is 400 nm (Abbe diffraction limit, with a numerical aperture of the 40x objective of 0.60 and a center wavelength of the bandpass filter of λ = 470 nm). Upon rotation of the sample in 90° steps, one can nicely follow the corresponding rotation of the horseshoe-shaped region. Most importantly, there are no changes in the sign and amplitude of the CD response of this region. Therefore, we conclude that contributions resulting from a combination of linear dichroism (LD) and linear birefringence (LB) in combination with any possible anisotropies of the CD microscopy setup can be safely excluded for length scales down to 400 nm. The finding is confirmed by the OD_L, OD_R, CD and g_{abs} spectra in the bottom row, which all yield results which are invariant upon rotation.

In addition, Supporting Figure 4.2 shows area-integrated spectra for OD_L, OD_R, OD_L–OD_R and g_{abs} of an arbitrary area of an F8BT:(+)-**2** thin film obtained using the CD microscope setup. There are virtually no changes in the CD response and the dissymmetry factor g_{abs} upon turning the thin film sample, meaning that the response of the samples is invariant under rotation. Thus, we can again exclude contributions resulting from a combination of LD and LB effects in our sample in combination with any possible anisotropies of the CD microscope optics. We previously observed very similar behavior for thin films of the intrinsically chiral copolymer c-PFBT. Such a behavior is a hallmark of multidomain supramolecular liquid crystalline order with statistical orientation of the individual domains.[9,10] There, it was also shown that such samples do not change their CD response when the sample is flipped.

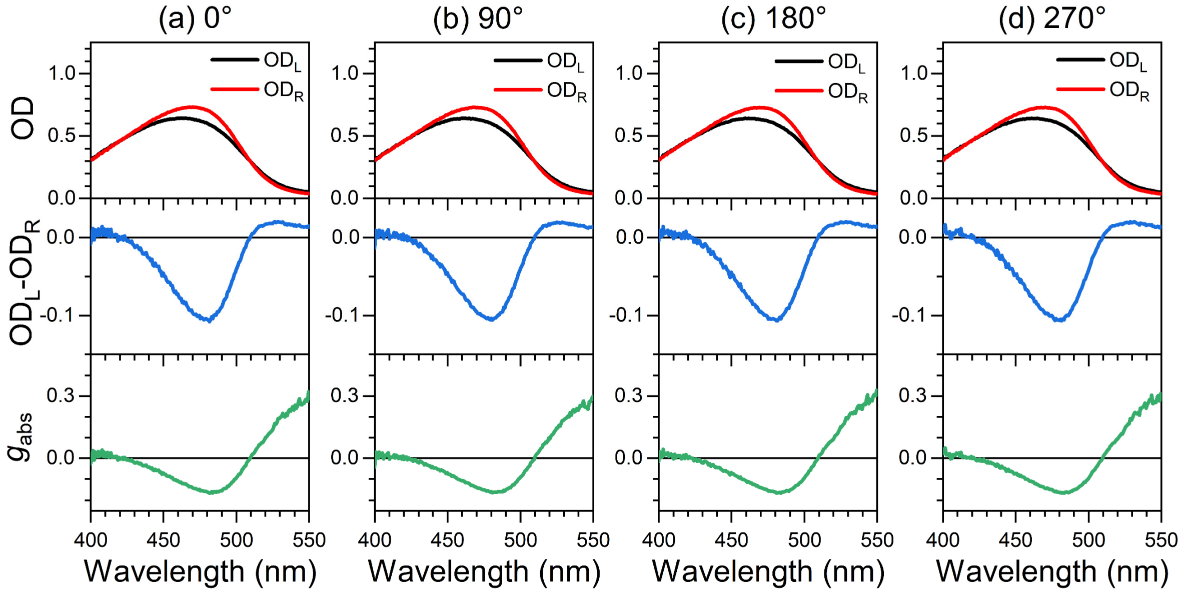

Supporting Figure 4.2. CD microscope spectra of an F8BT:(+)-2 thin film (33 wt% of the chiral additive) integrated over the entire field of view (210 × 160 μm^2) displaying the optical density for left- and right-circularly polarized light (OD_L (black line), OD_R (red line)), the CD spectrum (OD_L-OD_R, blue line) and the g_{abs} spectrum (green line). Spectra were recorded for the same film area by 90° stepwise rotation in anticlockwise direction. a) 0°. b) 90°. c) 180°. d) 270°.

Supporting Note 5.
DSC Measurements for Pure F8BT and an F8BT:(+)-2 Blend

In Supporting Figure 5.1, we show the heat flow $\dot{Q}$ measured in the DSC experiments as a function of temperature for pure F8BT (black) and the F8BT:(+)-**2** blend with 33 wt% of the chiral additive. Two heating runs for the temperature ranges 30–320 °C and 30–330 °C are shown as solid and dotted lines, respectively.

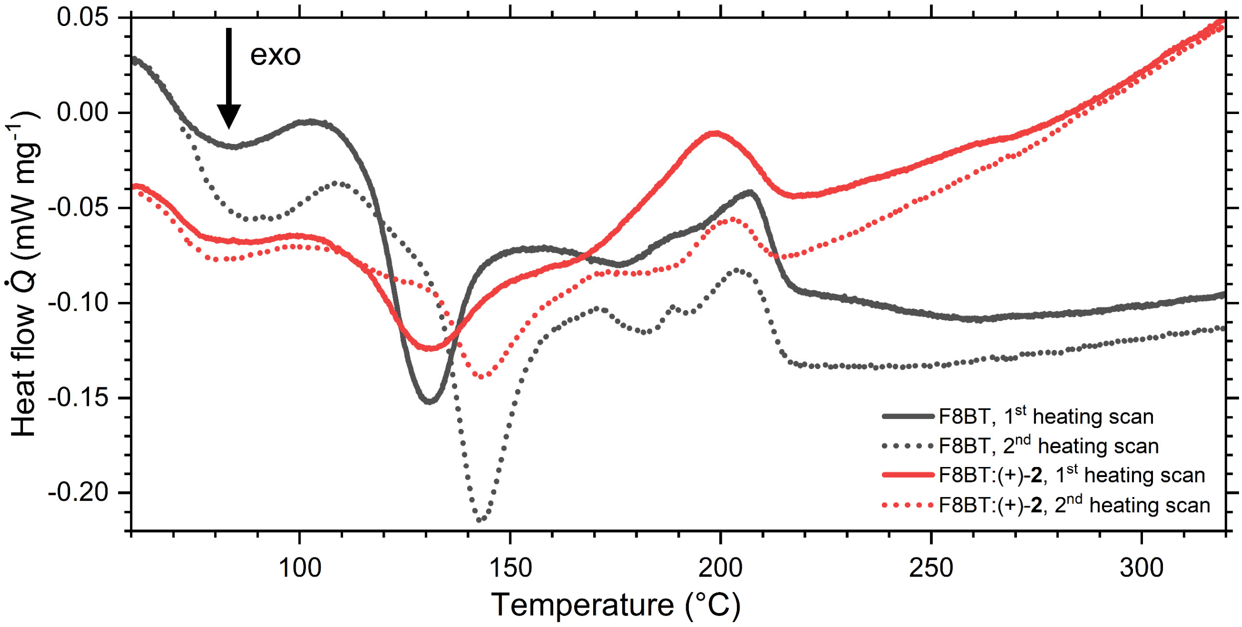

Supporting Figure 5.1. Comparison of the temperature-dependent heat flows for two heating scans of F8BT and F8BT:(+)-2. Solid lines: first heating scans, dotted lines: second heating scans, black: pure F8BT, red: F8BT:(+)-**2** blend with 33 wt% of the helicene-type chiral additive.

A further analysis was performed using the smoothed first derivative of the heat flow. Results for F8BT and the F8BT:(+)-**2** blend are shown in Supporting Figures 5.2 and 5.3, respectively. We start with the discussion of the first heating scan (top panels). An exothermic peak at 130 °C in the heat flow curve was attributed to the crystallization step from an amorphous glassy state to a nematic phase or rubbery state.[2] A Gaussian peak analysis provided a minimum in the first derivative at 122.3 and 121.6 °C for pure F8BT and the F8BT:(+)-**2** blend, respectively, which corresponds to the inflection point of the heat flow. It was therefore assigned to the glass transition temperature T_g. Most importantly, addition of the chiral helicene-type compound practically does not change the glass transition temperature of the polyfluorene copolymer. The melting range is indicated by the last local maximum in the heat flow curve. The zero crossing of the first derivative provides a melting temperature T_m of 206.7 and 198.5 °C for pure F8BT and the F8BT:(+)-**2** blend, respectively.

A similar analysis was performed for the second heating scan (bottom panels). Here, we obtained values of T_g = 138.1 (137.2) °C and T_m = 204.2 (202.6) °C for pure F8BT (the F8BT:(+)-**2** blend). We note that the T_g values for the second heating scan agree well with the range 135–140 °C reported by Donley et al.[2] However, for a direct comparison of F8BT and the F8BT:(+)-**2** blend, the first heating scan is probably more reliable, as we noted some decomposition of the helicene-type additive starting at about 200 °C.

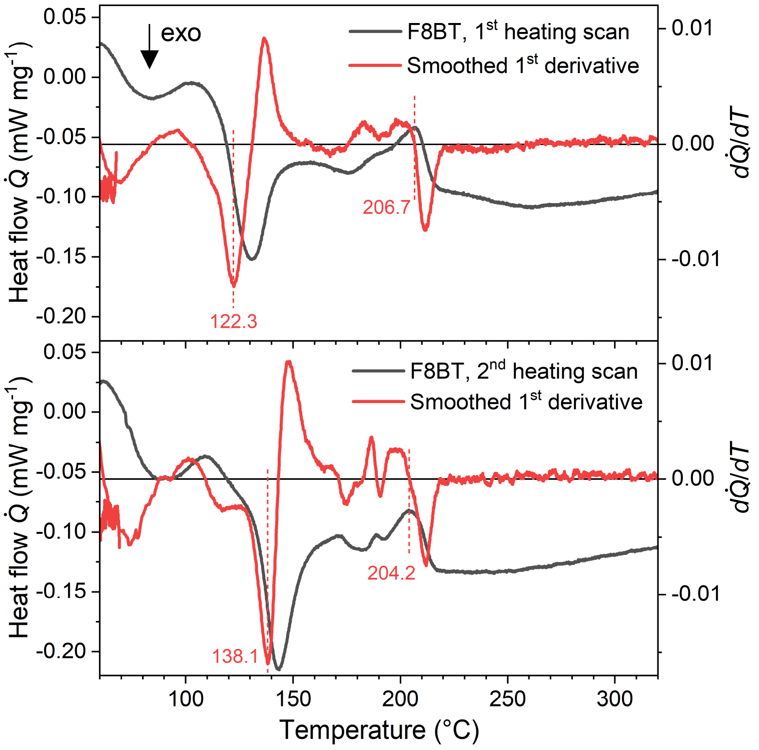

Supporting Figure 5.2. Analysis of transition temperatures for the first (top) and second (bottom) heating scan of F8BT from Supporting Figure 5.1. Black lines: temperature-dependent heat flow. Red lines: Smoothed first derivative.

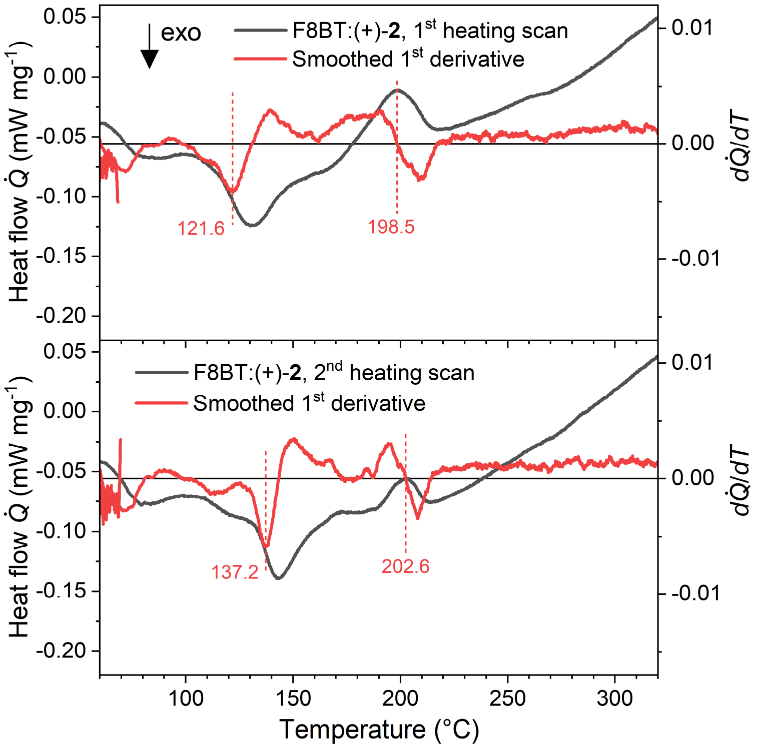

Supporting Figure 5.3. Same as in Supporting Figure 5.2, but for the F8BT:(+)-2 blend.

Supporting Note 6.
Ultrafast Electronic Relaxation of F8BT:(+)-2 and F8BT:(–)-2 Blends

Here, we discuss the dynamic processes underlying the TrCD signals in more detail. Representative kinetic traces averaged over the low-wavelength band of the TrCD bleach region (340–350 nm) of F8BT:(+)-2 and F8BT:(–)-2 were already presented in Figure 4d of the main manuscript. Both kinetic traces exhibit a fast decay over the first few picoseconds, which then slows down considerably, reaching a residual signal of about 15% at 1000 ps. These dynamics are directly correlated with the recovery of S_0 population, because the supramolecular TrCD response does not contain clear CD contributions from F8BT excited states.[4]

Importantly, the fast decay of the TrCD signal at early times is not compatible with the intrinsic lifetime of the S_1 exciton state. We determined this independently using time-correlated single photon counting (TCSPC) at very low S_1 exciton densities ($N(S_1) < 1 \times 10^{10}$ cm^{-3}), where processes involving two S_1 singlet excitons cannot occur because of the large exciton–exciton distance. Supporting Figure 6.1 shows such TCSPC curves recorded at magic angle after photoexcitation by femtosecond pulses at 410 nm for F8BT:(+)-**2** and F8BT:(–)-**2** thin films in panels a and b, respectively. The vertically polarized 410 nm pulses exclusively excite the F8BT copolymer, because the helicene-type chiral additive absorbs only below 390 nm (cf. Supporting Figure 10.1). Photons are detected at the magic angle (54.7°), and the time resolution of the setup is about 28 ps.

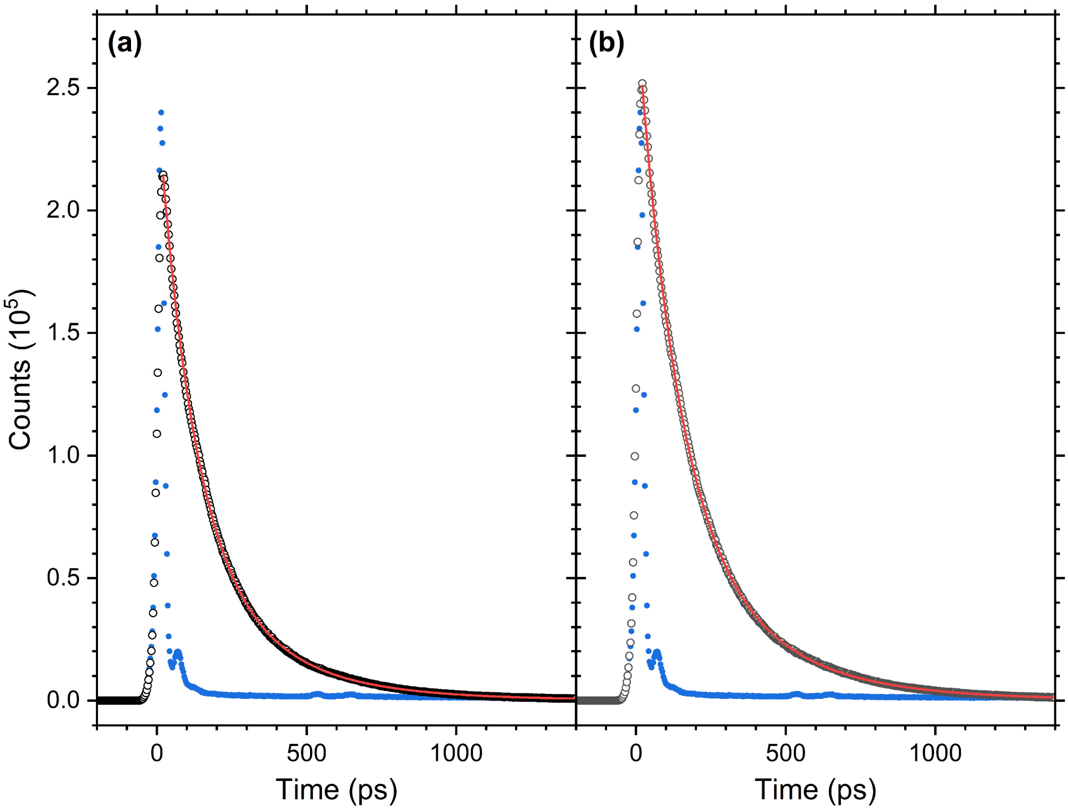

Supporting Figure 6.1. Time-resolved fluorescence experiments for the S_1 exciton state of F8BT in F8BT:(+)-2 and F8BT:(–)-2 thin films after photoexcitation at 410 nm. a) Kinetics of the F8BT:(+)-**2** thin film (open black circles) including a triexponential tail fit (red line) and the instrument response function (solid blue circles) recorded using a diluted colloidal SiO_2 nanoparticle solution. b) Same as in panel a, but for an F8BT:(–)-**2** thin film.

The decay of the photoluminescence intensity is well described by a triexponential function:

$$I(t) = \sum_{i=1}^{3} A_i \exp\left(-t / \tau_i\right) \qquad \text{(S6.1)}$$

The lifetimes τ_i and corresponding percentage amplitudes A_i were extracted from tail fits and are reported in Supporting Table 6.1. We also include fractional contributions to the photoluminescence intensity f_i (with i = 1, 2 or 3) which are defined as

$$f_i = \frac{A_i \cdot \tau_i}{\sum_{j=1}^{3} A_j \cdot \tau_j} \qquad \text{(S6.2)}$$

and the average lifetime $\langle \tau \rangle$, which is calculated as

$$\langle \tau \rangle = \frac{\sum_{j=1}^{3} A_j \cdot \tau_j^2}{\sum_{j=1}^{3} A_j \cdot \tau_j} \qquad \text{(S6.3)}$$

The multiexponential character of the decay is not unexpected, because individual F8BT chromophores in blends with the helicene-type additive experience quite different environments with varying contributions of F8BT–F8BT and F8BT–**2** interactions. The photoluminescence decays are dominated by two time constants: 119 and 286 ps for F8BT:(+)-**2** and 132 and 304 ps for F8BT:(–)-**2**. The 10–20 ps difference between the two films probably arises from slight variations in the film preparation conditions. The amplitude ratio is roughly 2:1 and the two components together amount to more than 99% of the total amplitude. The third time constant of about 1200 ps has only a contribution of 0.5% and could arise from a very small amount of a luminescing impurity in the polymer. The resulting average lifetimes obtained from eq. (S6.3) are 233 and 253 ps for the two different films. Note that this time scale is much slower than the initial decays observed in Figure 4d of the main manuscript, which are on the order of a few picoseconds.

Supporting Table 6.1. Kinetic parameters extracted from triexponential tail fits to the kinetics of the F8BT:(+)-2 and F8BT:(–)-2 thin films shown in Supporting Figure 6.1. Error bars for the time constants are based on a support plane analysis and are reported for a confidence interval of 95%.

	τ_i (ps)	A_i (%)	f_i (%)	$\langle \tau \rangle$ (ps)
		**F8BT:(+)-2**		
**Component 1**	119 ± 2	69.3	47.7	
**Component 2**	286 ± 4	30.3	49.6	233
**Component 3**	1240 ± 60	0.4	2.7	
		**F8BT:(–)-2**		
**Component 1**	132 ± 2	66.2	44.8	
**Component 2**	304 ± 5	33.3	52.3	253
**Component 3**	1204 ± 60	0.5	2.9	

A more elaborate kinetic description is therefore required to describe the kinetics in Figure 4d of the main manuscript for the blends consisting of F8BT and the helicene-type chiral additives (+)-**2**

and (–)-**2**. We utilize a simplified version of the kinetic model introduced recently for the relaxation of c-PFBT thin films:[4]

$$S_x \xrightarrow{k_x} S_1 \xrightarrow{k_1} S_0 \quad (S6.4)$$

$$S_x \xrightarrow{k_{CPx}} e^- + h^+ \quad (S6.5)$$

$$2\,S_1 \xrightarrow{k_{diff}} S_n + S_0 \quad (S6.6)$$

$$S_n \xrightarrow{k_n} S_1 \quad (S6.7)$$

$$S_n \xrightarrow{k_{CPn}} e^- + h^+ \quad (S6.8)$$

$$e^- + h^+ \xrightarrow{k_{rec}} S_0 \quad (S6.9)$$

In the following, we briefly summarize the key steps of the model:[4] Initially, a higher-lying singlet exciton state, denoted as S_x, is excited by the pump wavelength 320 nm. It decays with the rate constant $k_x = \tau_x^{-1}$ to S_1 singlet excitons, which relax further to S_0 with the rate constant $k_1 = \tau_1^{-1}$ (step S6.4). We associate τ_1 with the average lifetime $\langle \tau \rangle$ determined from the TCSPC measurements and use an averaged value of 243 ps for the F8BT copolymer in both films (cf. Supporting Table 6.1). As discussed previously, the S_x state can also directly dissociate into a charge pair state (CP, electron (e^-) plus hole (h^+)) according to step S6.5 (rate constant $k_{CPx} = \tau_{CPx}^{-1}$).[4,11]

Beside unimolecular singlet exciton relaxation, there are also singlet–singlet annihilation (SSA) channels, which are only efficient at high exciton number densities. One possibility is diffusive bimolecular SSA according to step S6.6. Here, two S_1 excitons diffuse along an F8BT chain and then react, once they reach a critical distance. This leads to the formation of a high-energy exciton species S_n and an S_0 ground state. Alternatively, SSA between two S_1 excitons may also take place by nonradiative Förster resonance energy transfer (FRET).[12,13] However, our previous study on c-PFBT showed that this is only a negligible channel.[4] Therefore, for the simple model applied here, the FRET process is not included. The excited species S_n can either decay to S_1 ($k_n = \tau_n^{-1}$, step S6.7) or produce a charge pair via step S6.8 with $k_{CPn} = \tau_{CPn}^{-1}$. Bimolecular recombination of the charge pair occurs on longer time scales ($k_{rec} = \tau_{rec}^{-1}$) and is described by step S6.9.

Contributions of the chiral additive to the spectral dynamics can be neglected, because the spectral CD signatures of films of these helicene-type molecules are distinctly different and much weaker than those of chiral F8BT arrangements.[14] However, the electronic interactions between the chiral additive and F8BT in addition to the F8BT–F8BT interactions might influence the relaxation time constants of F8BT. In addition, the overlap of the two absorption bands at the pump wavelength of 320 nm needs to be considered, because a fraction of the photons will be absorbed by the chiral additive and will not be available for the photoexcitation of F8BT. This can be evaluated by separating the steady-state absorption spectra of the blends into absorption contributions of the chiral additive and F8BT, leading to an effective absorbance of F8BT at 320 nm. Examples for this procedure are shown in Supporting Figure 10.1. The effective absorbance enters the determination of the initial F8BT exciton number densities, which were 1.12×10^{19} cm^{-3} for F8BT:(+)-**2** and 9.32×10^{18} cm^{-3} for F8BT:(–)-**2**.

In Supporting Figure 6.2, the TrCD signals at short times (panel a) and long times (panel b) are displayed in terms of the absolute exciton number densities. The TrCD kinetics monitor the recovery of the S_0 ground state population and do not show contributions from electronically excited states.

The experimental data for F8BT:(+)-**2** (open blue circles) and F8BT:(–)-**2** (open blue squares) therefore directly reflect the number density of F8BT in S_0. The kinetic model given by steps S6.4–S6.9 was implemented using the program package Tenua 2.1.[5] The kinetic parameters were optimized to simultaneously fit the TrCD kinetics of both films. Compared with the fits for the previously investigated c-PFBT system,[4] the rate constant k_{CPx} had to be increased to account for the larger quantum yield for CP formation from S_x. The rate constant k_{rec} for CP recombination was slightly reduced to describe the saturation behavior of the kinetic traces at long times better.

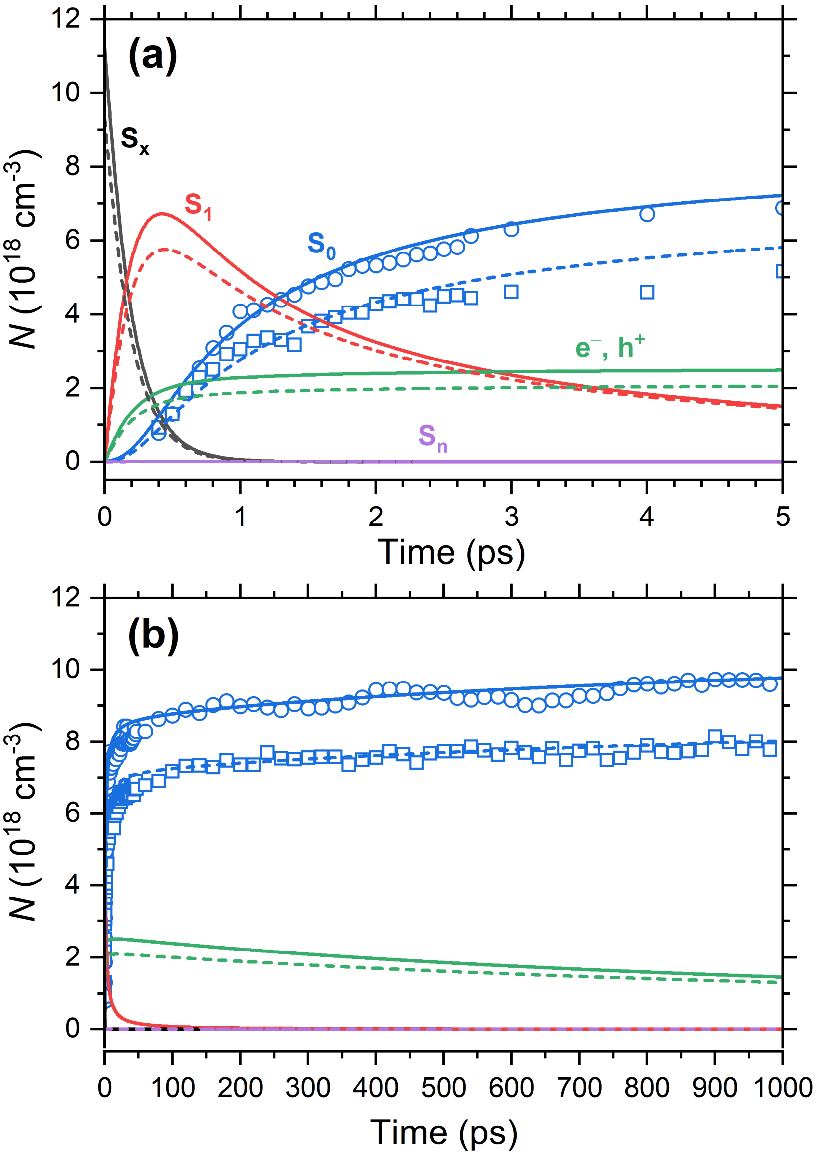

Supporting Figure 6.2. Kinetic modeling of the TrCD kinetics of F8BT:(+)-2 and F8BT:(–)-2 thin films. a) Results of the kinetic modeling for short time scales (up to 5 ps). Blue open circles and blue open squares: Recovery of S_0 population for the initial S_x exciton number densities 1.12×10^{19} cm^{-3} (F8BT:(+)-**2**) and 9.32×10^{18} cm^{-3} (F8BT:(–)-**2**), respectively, as experimentally determined by the TrCD measurements with corresponding solid and dashed blue fit lines obtained from the kinetic model; solid and dashed black, red, green and violet lines: number density of the S_x excitons, S_1 excitons, electron–hole pairs (e^-, h^+) and the S_n excitons, as obtained from the kinetic model. b) Same as in panel a, but for longer time scales up to 1 ns.

The kinetic model describes the changes in the S_0 population very well, both on short and long time scales, as shown by the solid blue lines (F8BT:(+)-**2**) and the dashed blue lines (F8BT:(–)-**2**). The fast recovery at early times (panel a) is predominantly due to diffusive S_1–S_1 exciton annihilation, where the S_1 exciton number densities are shown as solid and dashed red lines. The TrCD kinetics also provide information regarding the efficiency of CP formation from the highly excited S_x and S_n exciton states of F8BT. Taking the results from the best fit, we obtain a yield of 19% for charge pair formation from S_x and about 6% for the same process in the S_n state. The total yield of charge pair formation is therefore 25% and thus slightly larger than the 15% previously found in the case of c-PFBT thin films. Supporting Table 6.2 summarizes the parameters obtained from the optimized fit.

Supporting Table 6.2. Summary of parameters obtained from the kinetic analysis of the TrCD experiments for F8BT:(+)-2 and F8BT:(–)-2 upon photoexcitation at 320 nm.

Physical quantity (unit)	Value	Physical quantity (unit)	Value
$N_{0,\mathrm{F8BT:(+)\text{-}2}}(S_x)$ (cm^{-3})	1.12×10^{19}		
$N_{0,\mathrm{F8BT:(-)\text{-}2}}(S_x)$ (cm^{-3})	9.32×10^{18}		
$k_x(S_x \rightarrow S_1)$ (s^{-1})	4.40×10^{12}	τ_x (fs)	227
$k_{CPx}(S_x \rightarrow e^- + h^+)$ (s^{-1})	1.00×10^{12}	τ_{CPx} (ps)	2.22
$k_{x,total} = k_x + k_{CPx}$ (s^{-1})	5.40×10^{12}	$\tau_{x,total}$ (fs)	185
		Φ_{CPx} (%)	19
$k_1(S_1 \rightarrow S_0)$ (s^{-1})	4.12×10^{9}	τ_1 (ps)	243
$k_{diff}(2\,S_1 \rightarrow S_n + S_0)$ (cm^3 s^{-1})	1.10×10^{-7}		
$k_n(S_n \rightarrow S_1)$ (s^{-1})	3.8×10^{14}	τ_n (fs)	3
$k_{CPn}(S_n \rightarrow e^- + h^+)$ (s^{-1})	2.3×10^{13}	τ_{CPn} (fs)	40
$k_{n,total} = k_n + k_{CPn}$ (s^{-1})	4.0×10^{14}	$\tau_{n,total}$ (fs)	3
		Φ_{CPn} (%)	6
$k_{rec}(e^- + h^+ \rightarrow S_0)$ (cm^3 s^{-1})	3.0×10^{-10}		

Supporting Note 7.
Transient CPL Measurements Employing Excitation by a UV-LED

Supporting Figure 7.1 shows additional TrCPL experiments employing UV excitation at 273 nm by an unpolarized LED pulsed at 20 MHz. Here, the time resolution is much lower because of the longer LED pulse (FWHM 500 ps). Photoexcitation at 273 nm populates a higher singlet exciton state, which relaxes back to the luminescing S_1 exciton state on sub-picosecond time scales (cf. step (S6.4)). The results are consistent with those shown in Figure 5 of the main manuscript employing femtosecond laser excitation at 410 nm: We find a negative TrCPL signal for F8BT:(+)-**2** and a positive TrCPL signal for F8BT:(–)-**2**, (panel e) resulting in time-independent g_{lum} values of 0.30 and –0.38, respectively (panel f). The slight difference in g_{lum} values in the case of the F8BT:(–)-**2** thin film (cf. Figure 5f in the main manuscript) is likely due to different sample areas illuminated.

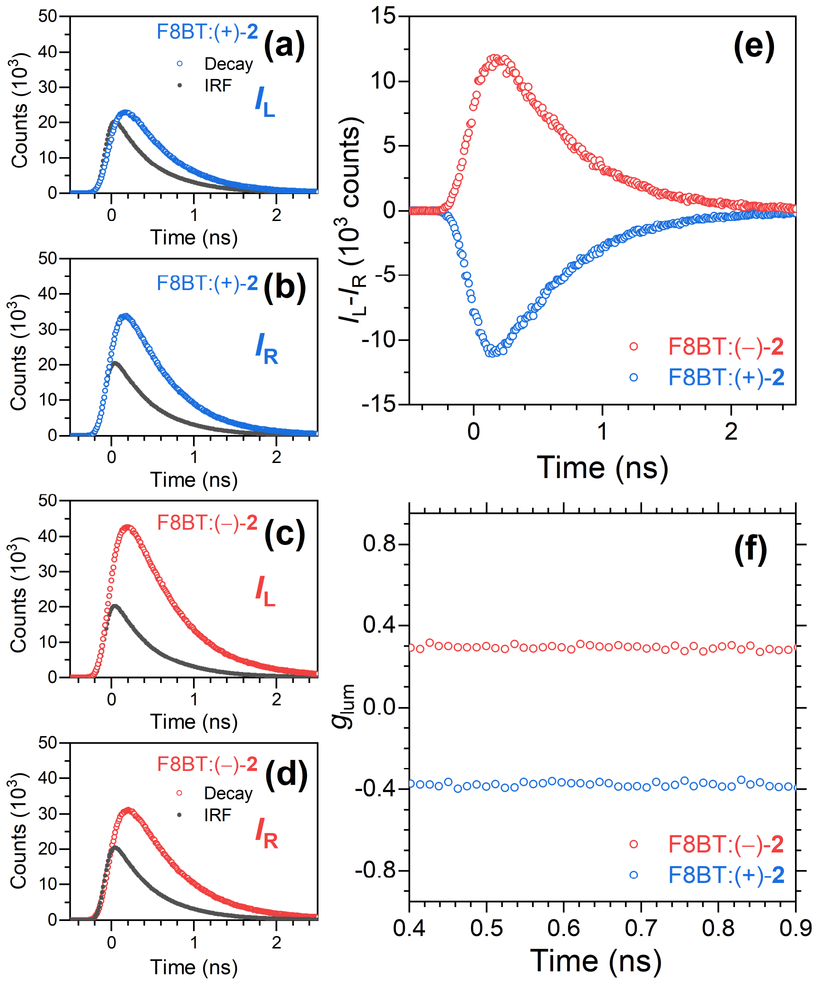

Supporting Figure 7.1. Time-resolved CPL spectroscopy for F8BT:(+)-2 and F8BT:(–)-2 thin films using a UV-LED for excitation (λ = 273 nm) and a bandpass filter at 530 nm for detection. a) Transient photoluminescence intensity I_L at left circular polarization for the F8BT:(+)-**2** film (open blue circles) and laser scattering signal I_L obtained from a colloidal SiO_2 nanoparticle suspension (solid black circles) providing the IRF of the setup. b) Same as in panel a, but showing the photoluminescence intensities I_R for right circular polarization. c) and d) Same as in panels a and b, but for the F8BT:(–)-**2** thin film and the same nanoparticle suspension. e) Resulting TrCPL signal I_L-I_R for the F8BT:(+)-**2** thin film (blue circles) and the F8BT:(–)-**2** thin film (red circles). f) Resulting time-resolved g_{lum} signal for the F8BT:(+)-**2** (blue circles) and F8BT:(–)-**2** (red circles) thin films.

Supporting Note 8.
Dependence of CD Signals on Film Thickness

Supporting Figures 8.1–8.3 show additional microscopy data for the dependence of the circular dichroism on film thickness. The experiments were performed on the same three films already discussed in the main manuscript (Figure 6) and serve to illustrate the spot-to-spot variation of the optical properties for the individual samples. In general, the average g_{abs} values agree very well for all samples, so the overall changes in the chiral response are minor. We used the variation of the g_{abs} values for the different film regions to estimate error bars in Figure 6e of the main manuscript.

In some regions of the films, however, there is a distinct increase of the width of the g_{abs} distributions, most prominently visible in Supporting Figures 8.2a and 8.3c. There are considerable variations in g_{abs}, as indicated by the fine mosaic structure, where bright and dark spots with sub-micrometer spacing indicate substantial changes in the dissymmetry factor on small length scales, whereas for other films this distribution is more uniform. We are currently performing systematic studies to identify the reason for these differences, which could for instance originate from local variations of the annealing temperature across the film area during film preparation.

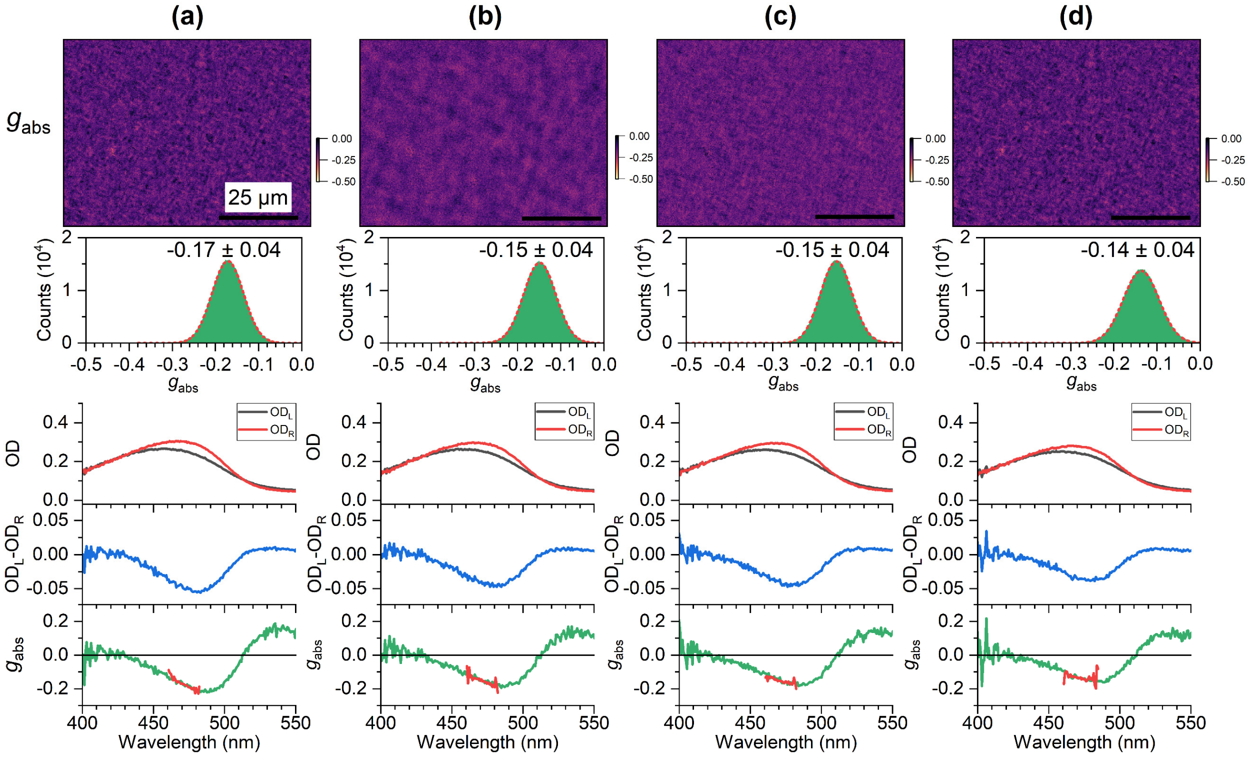

Supporting Figure 8.1. CD microscopy of a 103 nm thick F8BT:(+)-2 film. a) Microscope image (80 × 60 µm^2) for the dissymmetry parameter g_{abs} (top) with a histogram of g_{abs} values (green) including a Gaussian fit (dashed red line), determined over the entire field of view, and spectra integrated over the entire field of view (210 × 160 µm^2, lower half) displaying the absorption spectra for left- and right-circularly polarized light (OD_L (black line), OD_R (red line)), the CD spectrum (blue line) and the g_{abs} spectrum (green line; the thick red line indicates the spectral region selected by the bandpass filter used for CD imaging with a center wavelength of 470 nm). b-d) Same as in panel a, but at three other arbitrarily selected positions on the same film. The length of the black scale bar in each CD image corresponds to a distance of 25 µm.

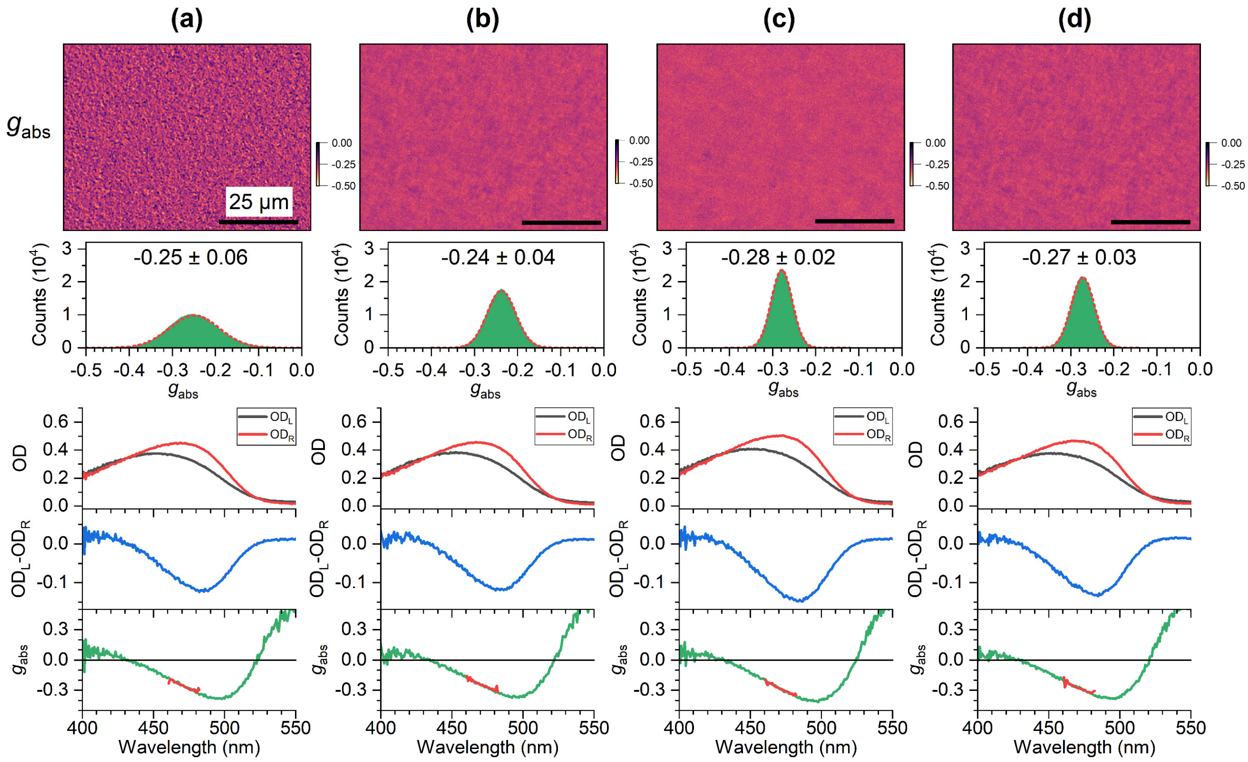

Supporting Figure 8.2. CD microscopy of a 159 nm thick F8BT:(+)-2 film. a-d) Microscope images for g_{abs}, g_{abs} distributions as well as absorption, CD and g_{abs} spectra, obtained using the same procedure and the same color coding as in Supporting Figure 8.1.

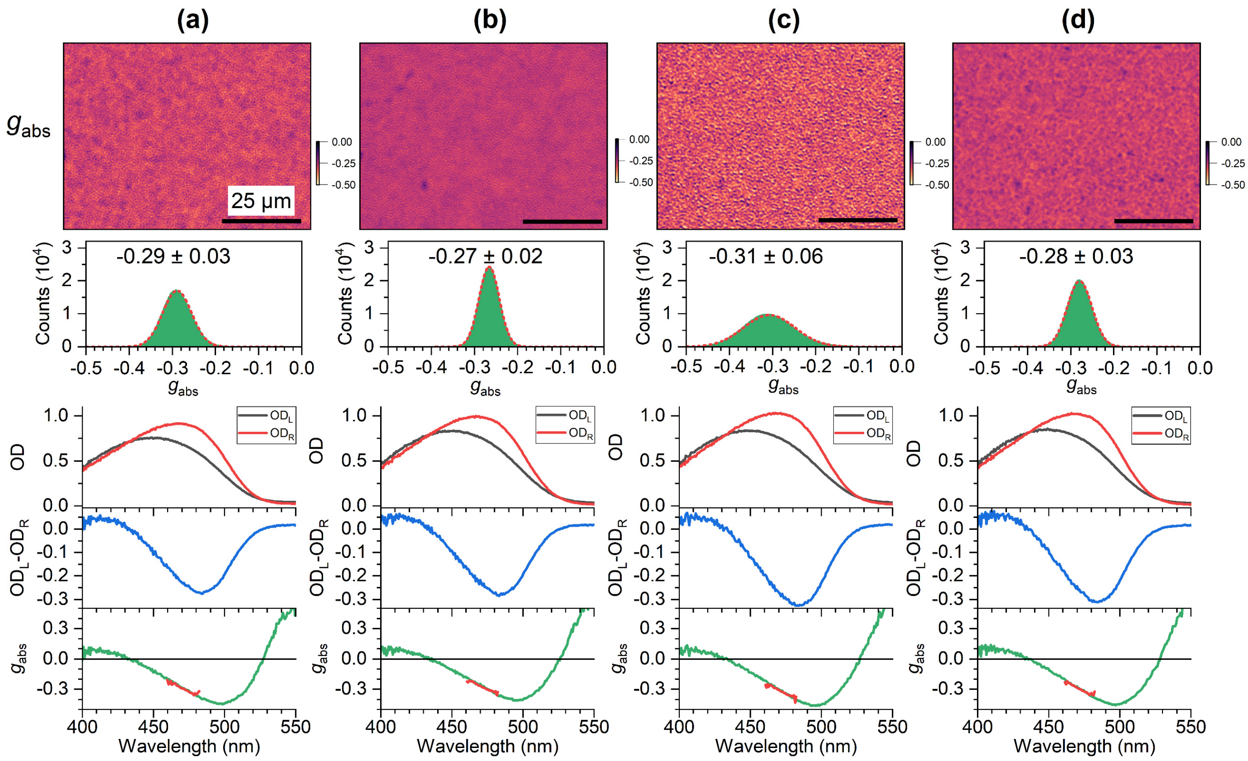

Supporting Figure 8.3. CD microscopy of a 356 nm thick F8BT:(+)-2 film. a-d) Microscope images for g_{abs}, g_{abs} distributions as well as absorption, CD and g_{abs} spectra, obtained using the same procedure and the same color coding as in Supporting Figure 8.1.

Supporting Note 9.
Reflectivity Correction for the Dissymmetry Parameter g_{abs}

Schulz et al. presented a formula to approximately account for reflection losses at thin films.[15] Their expression is derived from Stenzel's equation 7.9a for normal incidence on a thick slab neglecting interference effects and multiple reflections.[16] Following Schulz et al., the expression for the experimentally measured absorbance is

$$A^{\mathrm{exp}} = -\log_{10}\left(1-R\right)^2 + \frac{\alpha}{\ln(10)}d \tag{S9.1}$$

Here, α is the absorption coefficient (base *e*), *d* is the film thickness and *R* is the reflectivity obtained from Fresnel's formula for normal incidence

$$R = \left(\frac{n_2 - n_1}{n_2 + n_1}\right)^2 \tag{S9.2}$$

where n_1 is the refractive index of medium 1 and n_2 is the refractive index of medium 2. Thus, the corrected absorbance can be expressed as

$$A^{\mathrm{corr}} = \frac{\alpha}{\ln(10)}d = A^{\mathrm{exp}} + \log_{10}\left(1-R\right)^2 \tag{S9.3}$$

Consequently, the corrected dissymmetry factor is obtained as:

$$g_{\mathrm{abs}}^{\mathrm{corr}} = \frac{A_{\mathrm{L}} - A_{\mathrm{R}}}{A^{\mathrm{corr}}} = \frac{A_{\mathrm{L}} - A_{\mathrm{R}}}{A^{\mathrm{exp}} + \log_{10}\left(1-R\right)^2} \tag{S9.4}$$

Note that their expression is for a free-standing slab of material, which is not the experimental situation in our case. Because of the substantial difference in the refractive indices ($n_1 \approx 2.15$ at the peak of the g_{abs} spectrum of F8BT,[17] $n_2 \approx 1.0$ for air) the correction based on eq. (S9.4) will considerably overestimate the real correction, as it considers two interfaces of the copolymer thin film with air, whereas in our case we have the interfaces air–copolymer, copolymer–glass and glass–air. In any case, we obtain a value of –0.124 for the logarithmic correction term. As an example, for the three data points of the measurements dealing with the thickness dependence (violet solid circles in Figure 6e of the main manuscript), the corrected (uncorrected) absolute g_{abs} values for the three films are then, in increasing order of thickness: 0.408 (0.216), 0.598 (0.381) and 0.556 (0.443). Even after the correction, the g_{abs} value of the thinnest film is therefore substantially smaller than the g_{abs} values of the thicker films.

Supporting Note 10.
CD and AFM Experiments for Different F8BT:(+)-2 Blends

Supporting Figure 10.1 displays results from a systematic study of the chiral induction in the polyfluorene copolymer F8BT as a function of the weight percentage of the chiral additive (+)-**2** using identical spin-coating and annealing conditions. The steady-state CD spectrometer (Applied Photophysics Chirascan) used in this experiment was kindly made available by H. Ihmels (Organic Chemistry 2, University of Siegen). Employing 9 wt% of the additive (panel a) results in a peak CD signal of –0.025 (–830 mdeg) and a peak g_{abs} value of –0.05. An increase of the chiral response is observed when 17 wt% of (+)-**2** are used (panel b). Here, we obtain a peak CD value of –0.161 (–5300 mdeg) and peak g_{abs} value of –0.43. Using 33 wt% of the chiral additive leads to a further increase of the CD signal to –0.203 (–6700 mdeg), whereas the peak g_{abs} value is similar (–0.39). The results show that already 17% of the helicene-type compound (+)-**2** are sufficient to induce a substantial chiral response. The experiment also gives a good impression, how F8BT and the chiral additive contribute to the steady-state absorption spectrum of the blend (red and blue dashed lines in the top panels of Supporting Figure 10.1). To estimate the absorption contributions of (+)-**2** and F8BT in the blend, a scaled spectrum of a pure thin film of (+)-**2** (blue dashed line), which we measured previously,[14] was subtracted from the spectrum of the blend (black solid line), so that the characteristic peak of (+)-**2** at 369 nm disappeared in the resulting F8BT spectrum (red dashed line). Measurements of the absorption spectrum of pure F8BT show a smooth minimum in this wavelength range.

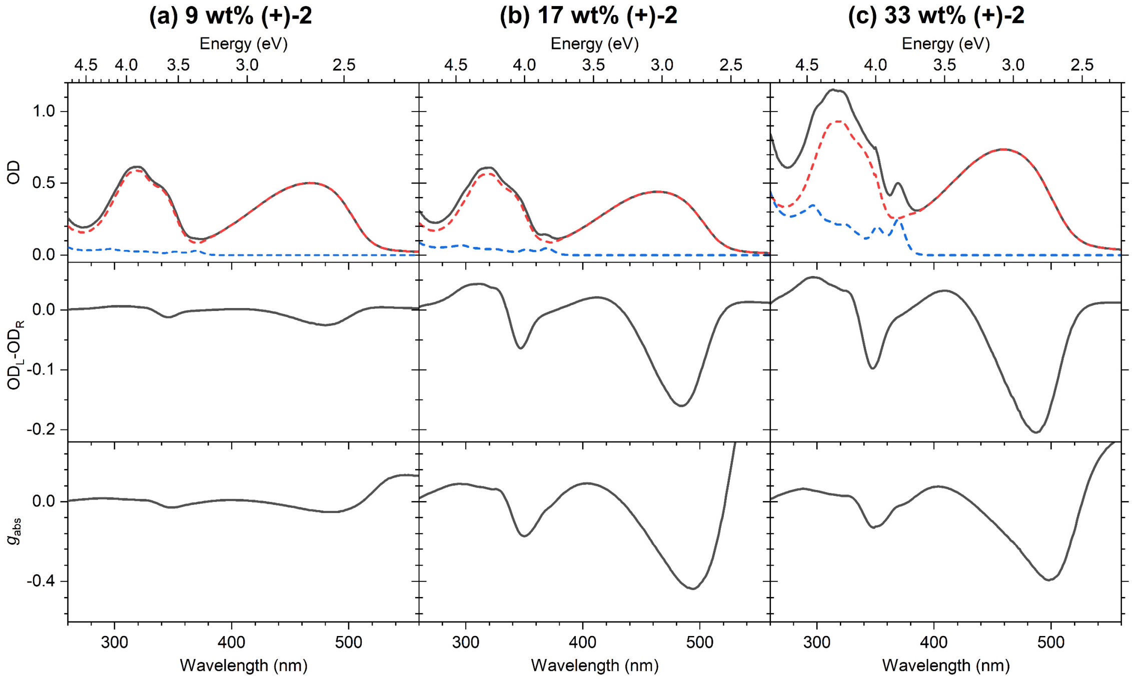

Supporting Figure 10.1. Chiral induction in the copolymer F8BT as a function of the weight percent of the chiral helicene-type additive (+)-2. a) For 9 wt% of (+)-**2**: Top panel: Steady-state absorption spectrum of F8BT:(+)-**2** (black solid line), scaled steady-state absorption spectrum of a pure (+)-**2** thin film (blue dashed line) and the difference between these two spectra (red dashed line), which should approximate the pure F8BT absorption in the blend; middle panel: CD spectrum, bottom panel: g_{abs} spectrum. b) and c) Same as in a, but for 17 wt% (+)-**2** and 33 wt% (+)-**2**, respectively. The values for the film thickness in panels a, b and c are 141, 120 and 235 nm, as measured by coherent acoustic phonon spectroscopy.

In addition, we carried out AFM and CD microscopy experiments for annealed pure F8BT and also F8BT:(+)-**2** thin films with varying amount of the chiral additive (9, 17 and 33 wt%) to check for a possible correlation between the surface morphology and the local CD activity of the thin films. The results are shown in Supporting Figure 10.2. The AFM images (top row) of the thin film surfaces show extended structures which could correspond to larger areas of fibrillar aggregates reported previously by Lakhwani and Meskers.[10] For the thin film with 33 wt% of the chiral additive, these structures appear to be more curved and elongated, with higher and lower regions being connected over longer distances. Values for the mean roughness S_a (RMS roughness S_q) were 3.02 (3.86), 5.88 (7.68), 4.54 (5.71) and 5.58 (6.76) nm for F8BT thin films with 0, 9, 17 and 33 wt% of the chiral additive, respectively. Thus, the roughness of the blends does not depend systematically on the amount of the chiral helicene-type additive in the film.

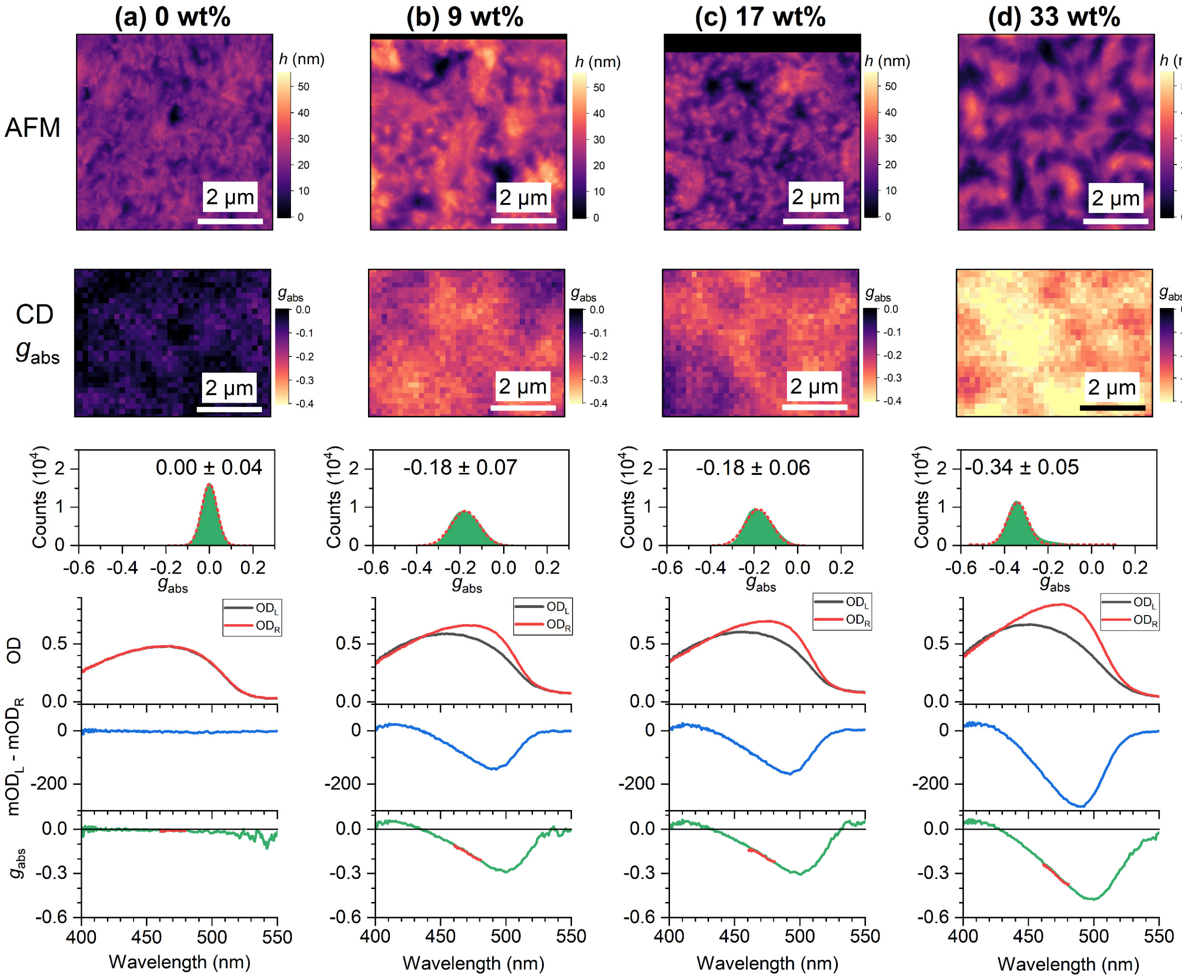

Supporting Figure 10.2. Comparison of atomic force microscopy and CD microscopy images for F8BT:(+)-2 films with different amounts of chiral additive. a) 0 wt% of the chiral additive (+)-**2**: AFM image with ca. 12 nm lateral resolution (top row), microscope image for the dissymmetry parameter g_{abs} with about 400 nm optical resolution (second row), histogram of g_{abs} values (green) with a Gaussian fit (dashed red line), determined over the entire field of view (third row), and spectra (bottom row) integrated over the entire field of view (210 × 160 μm^2) showing the optical density for left- and right-circularly polarized light (OD_L (black), OD_R (red)), the CD spectrum (blue) and the g_{abs} spectrum (green), with the thick red line indicating the spectral region selected by the bandpass filter (470 nm, FWHM 10 nm) employed for CD imaging. b-d) same as in panel a, but for thin films with 9, 17 and 33 wt% of the chiral additive, respectively.

In contrast to the AFM images, which are sensitive to the surface morphology, the CD images monitor the chiral optical response of the complete film in transmission. The CD microscope provides a diffraction-limited resolution of ca. 400 nm, whereas the AFM images shown here have a lateral resolution of about 12 nm. The CD microscopy images are therefore considerably more blurred than the AFM images. Taking this blurring into account, the larger structures observed by AFM and CD microscopy appear to be quite similar. The size of these larger domains is in the range 600–900 nm, similar to those seen in Supporting Figure S4.1.

Supporting References

[1] L. Guy, M. Mosser, D. Pitrat, J.-C. Mulatier, M. Kukułka, M. Srebro-Hooper, E. Jeanneau, A. Bensalah-Ledoux, B. Baguenard, S. Guy, *J. Org. Chem.* **2019**, *84*, 10870-10876.

[2] C. L. Donley, J. Zaumseil, J. W. Andreasen, M. M. Nielsen, H. Sirringhaus, R. H. Friend, J.-S. Kim, *J. Am. Chem. Soc.* **2005**, *127*, 12890-12899.

[3] D. Nečas, P. Klapetek, *Cent. Eur. J. Phys.* **2012**, *10*, 181-188.

[4] M. Morgenroth, M. Scholz, M. J. Cho, D. H. Choi, K. Oum, T. Lenzer, *Nat. Commun.* **2022**, *13*, 210.

[5] D. Wachsstock, Tenua 2.1 – the kinetics simulator for Java. http://bililite.com/tenua, **2007**.

[6] H. T. Grahn, H. J. Maris, J. Tauc, *IEEE J. Quantum Electron.* **1989**, *25*, 2562-2569.

[7] P. Ruello, V. E. Gusev, *Ultrasonics* **2015**, *56*, 21-35.

[8] M. Scholz, M. Morgenroth, M. J. Cho, D. H. Choi, T. Lenzer, K. Oum, *Struct. Dyn.* **2019**, *6*, 064502.

[9] D. Di Nuzzo, C. Kulkarni, B. Zhao, E. Smolinsky, F. Tassinari, S. C. J. Meskers, R. Naaman, E. W. Meijer, R. H. Friend, *ACS Nano* **2017**, *11*, 12713-12722.

[10] G. Lakhwani, S. C. J. Meskers, *J. Phys. Chem. A* **2012**, *116*, 1121-1128.

[11] M. Morgenroth, M. Scholz, T. Lenzer, K. Oum, *J. Phys. Chem. C* **2020**, *124*, 10192-10200.

[12] T. Förster, *Ann. Phys.* **1948**, *437*, 55-75.

[13] T. Förster, *Discuss. Faraday Soc.* **1959**, *27*, 7-17.

[14] M. Morgenroth, M. Scholz, L. Guy, K. Oum, T. Lenzer, *Mol. Phys.* **2021**, *119*, e1959072.

[15] M. Schulz, J. Zablocki, O. S. Abdullaeva, S. Brück, F. Balzer, A. Lützen, O. Arteaga, M. Schiek, *Nat. Commun.* **2018**, *9*, 2413.

[16] O. Stenzel, *The Physics of Thin Film Optical Spectra - An Introduction*, Springer Series in Surface Sciences (Vol. 44), Springer, Cham, **2016**.

[17] M. Campoy-Quiles, G. Heliotis, R. Xia, M. Ariu, M. Pintani, P. Etchegoin, D. D. C. Bradley, *Adv. Funct. Mater.* **2005**, *15*, 925-933.
